# Gestational Diabetes Mellitus: The Crosslink among Inflammation, Nitroxidative Stress, Intestinal Microbiota and Alternative Therapies

**DOI:** 10.3390/antiox11010129

**Published:** 2022-01-07

**Authors:** Elaine Luiza Santos Soares de Mendonça, Marilene Brandão Tenório Fragoso, Jerusa Maria de Oliveira, Jadriane Almeida Xavier, Marília Oliveira Fonseca Goulart, Alane Cabral Menezes de Oliveira

**Affiliations:** 1 Institute of Chemistry and Biotechnology, Federal University of Alagoas, Maceio 57072-970, Alagoas, Brazil; elaine.mendonca@fanut.ufal.br (E.L.S.S.d.M.); marilene.tenorio@iqb.ufal.br (M.B.T.F.); jerusa.amorim@iqb.ufal.br (J.M.d.O.); jadrianexavier@iqb.ufal.br (J.A.X.); 2 College of Nutrition, Federal University of Alagoas, Maceio 57072-970, Alagoas, Brazil

**Keywords:** insulin resistance, pregnancy, pro-inflammatory cytokines, reactive oxygen and nitrogen species, dysbiosis, natural products

## Abstract

Gestational diabetes mellitus (GDM) is characterized by a set of metabolic complications arising from adaptive failures to the pregnancy period. Estimates point to a prevalence of 3 to 15% of pregnancies. Its etiology includes intrinsic and extrinsic aspects of the progenitress, which may contribute to the pathophysiogenesis of GDM. Recently, researchers have identified that inflammation, oxidative stress, and the gut microbiota participate in the development of the disease, with potentially harmful effects on the health of the maternal-fetal binomial, in the short and long terms. In this context, alternative therapies were investigated from two perspectives: the modulation of the intestinal microbiota, with probiotics and prebiotics, and the use of natural products with antioxidant and anti-inflammatory properties, which may mitigate the endogenous processes of the GDM, favoring the health of the mother and her offspring, and in a future perspective, alleviating this critical public health problem.

## 1. Introduction

The gestational period corresponds to a physiologically natural state with organic adaptations of the mother to meet the maternal-fetus-placental needs. It includes adjustments to the cardiovascular, hematological, renal, pulmonary, and metabolic systems. Furthermore, the pregnancy cycle is a state with intense nitroxidative and inflammatory stresses, which play an essential role in embryonic-fetal implantation and development and in placental function [1,2,3,4].

Failures in these adaptive processes can lead to pregnancy-specific disease states, such as gestational diabetes mellitus (GDM), a metabolic disorder that develops from carbohydrate intolerance due to resistance or decreased sensitivity to the action of insulin, which leads to maternal hyperglycemia [5,6]. Reduced insulin sensitivity or resistance can also be intensified by an imbalance between reactive oxygen and nitrogen species (RONS) and pro-inflammatory cytokines, particularly concerning their excessive production and a defect in antioxidant and anti-inflammatory defenses [3,7,8].

It is noteworthy that the intestinal microbiota plays a crucial role in response to insulin sensitivity and in underlying inflammatory processes. Considering the microbiota as a therapeutic target in the GDM in addition to the benefits inherent to intestinal health, it also strengthens and intensifies the antiglycant, antioxidant, and anti-inflammatory properties of some bioactive compounds [9,10,11]. A possible relationship among the bioavailability, bioactivity, and effects of some bioactive compounds present in natural products to the intestinal microbiota, starting by bacterial conversion, is suggested [12].

In this context, the interactions listed above associated with the imbalance of carbohydrate homeostasis, present in the GDM, can trigger irreversible complications in the short and long terms for the maternal-fetal binomial, being considered an essential public health problem [3,7]. However, the available strategy for managing the GDM has been limited when considering only lifestyle changes and the use of insulin or oral antidiabetics, although there is universal recognition of the importance of using euglycemic, antioxidant, and anti-inflammatory compounds [7,13,14].

Among these agents, natural products have stood out, with properties that allow for modulating the intestinal microbiota, by regulation of the composition of intestinal bacteria and their metabolites. They may also improve the synthesis of short-chain fatty acids (SCFA) necessary in health and disease processes. Two particular mechanisms, activation of G-protein-coupled receptors (GPCRs) and inhibition of histone deacetylases (HDACs), can alter chemotaxis, RONS, proliferation, and cell function, reducing both the permeability among enterocytes and the production of lipopolysaccharides (LPS) [15,16,17].

Given this context, this review aims to critically analyze the reported pieces of evidence about the GDM, emphasizing inflammatory and nitroxidative stress aspects related to pathophysiology. In addition, the involvement of the intestinal microbiota and alternative therapeutic innovations can be used in the future to attenuate this critical public health problem by mitigating adverse maternal and perinatal outcomes.

## 2. Methods

This narrative literature review was carried out from September to November 2021. The electronic databases considered for the search of articles were Pubmed, Web of Science, and Science Direct. Two independent reviewers performed all steps of the review. There was no use of search filters (language, year of publication, or type of article). The MESH terms for the construction of the search strategy were selected from the Pubmed database, considered here as a reference. Each topic in this review had specific MESH terms, according to their respective objectives. All identified articles that included updated information on the GDM were considered eligible.

### 2.1. MESH Terms

#### 2.1.1. GDM

The MESH terms used for GDM were (“gestational diabetes mellitus” OR “gestational hyperglycemia” OR “Diabetes, Pregnancy-Induced” OR “Pregnancy in Diabetic” OR “Glucose intolerance during pregnancy” OR “Gestational glucose intolerance”) AND (“description” OR “characterization” OR “definition” OR “prevalence” OR “Screening” OR “Diagnosis” OR “etiology” OR “risk factors” OR “adverse outcomes”).

#### 2.1.2. Inflammatory Aspects in GDM

The MESH terms used for inflammatory aspects in GDM were (“gestational diabetes mellitus” OR “gestational hyperglycemia” OR “Diabetes, Pregnancy-Induced” OR “Pregnancy in Diabetic” OR “Glucose intolerance during pregnancy” OR “Glycation End Products, Advanced” OR “Gestational glucose intolerance” OR “Advanced Glycation End-products in pregnancy” OR “AGEs in pregnancy”) AND (“inflammatory pathways” OR “inflammation” OR “NF-kappa B” OR “NF-kB” OR “STAT Transcription Factors” OR “STAT3”) AND (“physiopathology”).

#### 2.1.3. Nitroxidative Stress in GDM

The MESH terms used for nitroxidative stress in GDM were (“gestational diabetes mellitus” OR “gestational hyperglycemia” OR “Diabetes, Pregnancy-Induced” OR “Pregnancy in Diabetic” OR “Glucose intolerance during pregnancy” OR “Glycation End Products, Advanced” OR “Gestational glucose intolerance” OR “Advanced Glycation End-products in pregnancy” OR “AGEs in pregnancy”) AND (“Oxidative Stress” OR “Nitro-Oxidative Stress” OR “polyol pathway” OR “Protein Kinase C” OR “PKC” OR “hexosamine pathway” OR “NADPH oxidase” OR “xanthine oxidase” OR “mitochondrial pathway”) AND (“physiopathology”).

#### 2.1.4. Intestinal Microbiota and GDM

The MESH terms used for intestinal microbiota and GDM (“gestational diabetes mellitus” OR “gestational hyperglycemia” OR “Diabetes, Pregnancy-Induced” OR “Pregnancy in Diabetic” OR “Glucose intolerance during pregnancy” OR “Glycation End Products, Advanced” OR “Gestational glucose intolerance” OR “Advanced Glycation End-products in pregnancy” OR “AGEs in pregnancy”) AND (“Prebiotics” OR “probiotic” OR “Synbiotics” OR “gut microbiota” OR “Gastrointestinal Microbiome” OR “Microbiota” OR “microbiota modulation”) AND (”Antidiabetic” OR “antiglycant” OR “antioxidant” OR “anti-inflammatory” OR “Anti-hyperglycemic”).

#### 2.1.5. Alternative Therapies in GDM

##### Randomized Clinical Trials

The MESH terms used for alternative therapies in GDM conducted as randomized clinical trials were (”gestational diabetes mellitus” OR “gestational hyperglycemia” OR “Diabetes, Pregnancy-Induced” OR “Pregnancy in Diabetic” OR “Glucose intolerance during pregnancy” OR “Glycation End Products, Advanced” OR “Gestational glucose intolerance” OR “Advanced Glycation End-products in pregnancy” OR “AGEs in pregnancy”) AND (“complementary therapies” OR “alternative medicine” OR “alternative therapies” OR “phytotherapy” OR “nutrition therapy” OR “natural products” OR “bioactive compounds”) AND (”Antidiabetic” OR “antiglycant” OR “antioxidant” OR “anti-inflammatory” OR “Anti-hyperglycemic”).

##### Experimental Studies in Animal Models

The MESH terms used for alternative therapies in GDM conducted experimental studies in animal models were (“gestational diabetes mellitus” OR “gestational hyperglycemia” OR “Diabetes, Pregnancy-Induced” OR “Pregnancy in Diabetic” OR “Glucose intolerance during pregnancy” OR “Glycation End Products, Advanced” OR “Gestational glucose intolerance” OR “Advanced Glycation End-products in pregnancy” OR “AGEs in pregnancy”) AND (“Phytotherapy” OR “medicinal plants” OR “Plant Extracts” OR “natural products” OR “extrats”) AND (“Antidiabetic” OR “antiglycant” OR “antioxidant” OR “anti-inflammatory” OR “Anti-hyperglycemic”).

## 3. GDM

GDM reflects a set of endocrine complications arising from adaptive organ failure, considered the most common metabolic disorder of the pregnancy period [5]. Its recognition occurs through the identification of spontaneous hyperglycemia, during pregnancy, and without precedents [5,18]. Global estimates indicate that gestational hyperglycemia affects an average of 16.2% of pregnancies, among which 86.4% are due to GDM (Box 1) [19].

Box 1 **Global and regional estimates of gestational hyperglycemia.**

**Global Prevalence**

**Prevalence by Region**
Hyperglycemia in pregnancy16.2%Africa 10.4% Western Pacific 12.6%South America and Central America 13.1%North America and Caribbean 14.6%Europe 16.2%Middle East and North Africa 21.8%South East Asia 24.2%**Source:** Adapted from International Diabetes Federation [19].

The regions identified as those with the highest prevalence for the GDM have been low- and middle-income countries, where access to maternal health services is usually precarious or limited, with the Asian region being the one with the highest percentage (24.2%) [19,20]. The disparities observed in the global epidemiological panorama may be due to the diagnostic criteria used, since there is still no consensus among organizations regarding the standardization of classification and diagnosis for the disease [19].

### 3.1. Screening and Diagnosis

From the first reports to the present, there is no consensus between health organizations and entities regarding the diagnostic criteria for the GDM [21,22,23,24,25,26,27,28]. Despite the divergences, the most commonly accepted criterion is the one of International Association of Diabetes in Pregnancy Study Group (IADPSG) [27], which was based on the study Hyperglycemia and Adverse Pregnancy Outcomes (HAPO) [25,29], establishing that pregnant women with changes in glucose parameters, identified during the 24th–28th gestational weeks, could be diagnosed with GDM (Box 2) [27].

Box 2 **Diagnostic and screening criteria for gestational diabetes mellitus (GDM).**

**Criteria**

**Time Course**

**Fasting Glucose (mg/dL)**

**Glucose Overload**

**Oral Glucose Tolerance Test (mg/dL)**

**1 h**

**2 h**

**3 h**
O’Sullivan & Mahan (1964) [21]Detected at any time during pregnancy90100 g of glucose165 145 125 O’Sullivan & Mahan (1964) [21] adapted by National Diabetes Data Group (NDDG) (1979) [22]105100 g of glucose190 165145Carpenter & Coustan (1982) [23]95100 g of glucose180 155 140 World Health Organization (WHO) (1999) [26]12675 g of GlucoseNot measured140Not measuredInternational Association of Diabetes in Pregnancy Study Group (IADPSG (2010) [27]24–28 gestational weeks 9275 g of Glucose180153

The American Diabetes Association (ADA), World Health Organization (WHO), Endocrine Society, and the International Federation of Gynecology and Obstetrics (IFGO) recommended the use of the criteria proposed by IADPSG [30,31,32,33]. However, it is noteworthy that the diagnostic criteria established by the IADPSG expands the population of pregnant women diagnosed with GDM, reflecting health costs, in addition to not considering the risk factors in its screening, which could be a limiting factor [28]. In Brazil, for instance, when comparing studies based on methodologies with different diagnostic criteria, it can be observed that the prevalence for GDM was about twice as high using the IADPSG criteria (18.0%) compared with the ones based on the first criterion established by the WHO (7.6%) [34,35].

This increase in the prevalence of GDM may impact the country’s economy. In this sense, IFGO recommended that, in the presence of financial feasibility and technical availability, the IADPSG criteria should be used. However, it is the responsibility of each region to analyze and propose the adoption of the best diagnostic criteria for GDM, according to available resources [32]. The recent epidemiological and nutritional transition had negative impacts on the profile of nutritional status, eating habits, and sedentary lifestyle of the population. The adoption of screening of the risk factors for GDM should be considered, especially for health services with financial and technical limitations [28,32].

### 3.2. Etiology

GDM has well-documented risk factors, which include maternal chronological age, family history of type 2 diabetes mellitus (T2DM), genetics, race/ethnicity, geography, socioeconomic status, DMG overweight, western-like diet, sedentary lifestyle, exposure to chemicals, polycystic ovary syndrome, vitamin D deficiency, and adverse birth conditions of the mother [3,36,37]. These risk factors are directly or indirectly associated with impaired β-cell function and/or insulin sensitivity. The Box 3 lists the mechanisms of action possibly related to the development of GDM.

Box 3 **Risk factors attributed to the development of GDM.**

**Risk Factor**

**Mechanism of Action**

**Reference**

**Advanced chronological age**

**(>35 years old)**

▪Processes inherent to senescence:
-During aging, the body can lose efficiency in repairing flaws or adapting to organic changes; thus, a late pregnancy can culminate in adaptive metabolic failure processes, contributing to resistance or decreased insulin sensitivity.

[38,39]
**Family history**

▪Family history for T2DM and the development of GDM:
-During a normal pregnancy, more specifically in the third trimester, to meet the needs of fetal growth and development, maternal lipid metabolism changes (↓the activity of lipases, resulting in the increase (↑) of triglycerides (TG) and decrease (↓) of high-density lipoprotein cholesterol (HDL-c). After an adaptive period, they return to normal levels. However, there are failures in the feedback processes in pregnant women who develop GDM, which also occurs in T2DM.

[40,41]
**Genetic factors**

▪Genetic modifications shared between T2DM and GDM:
-Some genes common in both T2DM and GDM correspond to genetic mutations related to decreased insulin secretion, such as genes CDK5 regulatory subunit-associated protein 1-like 1 (CDKAL1, cyclin-dependent kinase inhibitor 2A/2B (CDKN2A/2B), and hematopoietically expressed homeobox (HHEX).

[40,41,42]
▪GDM-related genetic mutations:
-Genetic mutations in some specific genes are related to the development of GDM, such as the following genes: transcription factor 7-like 2 (TCF7L2), CDKAL1, Transcription factor 2 (TCF2), Fat mass- and obesity-associated gene (FTO), CDKN2A/2B, HHEX, Insulin-like growth factor 2 MRNA binding protein 2, Solute carrier family 30 member 8 gene (IGF2BP2), and SCL30A8.-Some women, although uncommon among pregnant women with GDM, have genetic variants that are monogenic forms of diabetes, including genes for subtypes maturity onset diabetes of the young (MODY).
[40,42,43]
**Race/**

**Ethnicity**

▪Hispanic pregnant women would have greater chances of developing GDM, when compared with non-Hispanic ones, which can be considered a confonding factor when the geographic characteristics are inserted.
[44,45]
**Geographic features**

▪Depending on the territorial socio-economic limitation, which comprises government and population, data on the GDM may be under or overestimated since they depend on the diagnostic criteria adopted for screening the GDM, and, thus, on the financial and technical resources available in the country/region.
[28,32]
**Socio-economic**

▪Precarious socio-economic conditions, such as low income and education, and unemployment, may be related to worse gestational conditions, ↑ the risk for the development of GDM due to poor quality maternal care.
[45,46]
**Overweight**

▪Adipose tissue:
-It synthesizes adipokines, which can directly influence the production of pro-inflammatory cytokines (interleukin 1β (IL-1β), nterleukin 6 (IL-6), and Tumor necrosis factor α (TNF-α), and contribute to the increase of serum levels of C-reactive protein (CRP) and RONS. These factors favor the activation of the inflammatory cascade and, consequently, deregulate organic homeostasis, which may exacerbate the factors involved in the physiopathogenesis of GDM.

[3,47]
▪Positive energy balance:
-Caloric intake above daily needs, associated or not with a sedentary lifestyle, has an essential impact on insulin resistance, favoring the endogenous environment for the development of GDM.

[48,49]
**Westernized diet**

▪Dietary profile with high intake of red meat, sausages and ultra-processed products, refined products, sweets, pasta, and fried foods, also intensifies the mechanisms of insulin resistance, in addition to contributing to the underlying inflammatory process.
[37,50]
**Sedentary lifestyle**

▪The practice of physical activity reduces the chances of developing GDM by up to 46%, since a sedentary lifestyle, in turn, increases nitroxidative and inflammatory stress, and intensifies insulin resistance.
[51,52]
**Exposure to chemicals**

▪Perfluorooctanoic acid (PFOA)—commonly found in cleaning products, some types of containers and packaging):
-Studies in animal models have found that their contact with offspring could, in a single gestational exposure, have potential effects on postnatal growth and development, causing a delay on them. Furthermore, there is evidence that it can be transmitted through lactation, causing harmful impacts to the health of the offspring.-In humans, it was possible to identify a positive association between serum PFOA concentrations, with cholesterol, TG, and uric acid, which are related to pro-inflammatory pathways, and insulin resistance

[53,54,55,56,57,58]
▪Tobacco and alcohol:
-Independent risk factors for GDM, since its consumption may contribute to the endogenous increase in oxidative stress, inflammation, hyperglycemia and insulin resistance, although the exact mechanism of action has not yet been fully elucidated.

[59,60,61]
**Polycystic Ovary Syndrome (POS)**

▪Endocrine-metabolic disease that involves multiple hormonal changes related to female infertility, with symptoms such as insulin resistance, one of the most frequently observed, since approximately 50% of women with POS develop GDM during pregnancy.
[62,63]
**Vitamin D Deficiency**

▪Both vitamin D and parathormone (PTH) contribute to calcium (Ca) homeostasis. Vitamin D is responsible for the viability of the intestinal absorption of Ca, while PTH for maintaining Ca homeostasis in face of its deficiency. When serum Ca is at suboptimal concentrations, PTH stimulates Ca reabsorption from bone stores, and renal reabsorption, which could increase the risk of GDM, mediated by insulin resistance.
[64,65]
**Adverse birth conditions of the mother**

**(Fetal program)**

Mothers who were born in suboptimal conditions, such as premature, with Low birth weight (LBW), or small for gestational age (SGA), could trigger GDM in the pregnancy period, a theory known as fetal programming, postulated by Barker.Changes in somatic growth due to the shortage of nutrients in the pregnancy period lead to damage to the hypothalamus/growth hormone; (GH)/Insulin-like growth (IGF-1) axis. A deficit in the morphology of target organs, such as the pancreas, reduces it in size and affects the function of pancreatic β-cells, culminating in the deficiency in insulin production.
▪These conditions can lead to transgenerational effects, as a vicious cycle, causing serious consequences to public health.
[66,67,68,69,70]

### 3.3. Maternal and Perinatal Outcomes in the GDM

The effects of GDM on maternal blood glucose are usually attenuated after removal of the placenta and return of serum hormone levels. However, pregnant women affected by GDM have an increased risk in the course of pregnancy for recurrent urinary infections, ketoacidosis, prolonged labor (difficulty in fetal passage through the vaginal canal, increasing the risk of using forceps) or cesarean, perineal lacerations or ruptures, uterine atony (condition in which the uterus cannot perform adequate contraction, with the possibility of postpartum hemorrhage), and uterine rupture (particularly in pregnant women with a previous history of cesarean section) [6,71]. After the pregnancy period, these pregnant women have a seven-fold risk for the future development of T2DM and cardiovascular diseases (CVD) in addition to a higher rate for obesity and metabolic syndrome [3,7,8].

As mentioned above, GDM can also cause complications to the fetus in an immediate and/or future perspective. Regarding immediate adverse outcomes (short term), it is possible to observe an increased risk for macrosomic birth (>4.000 g) or large for gestational age—LGA (relationship between birth weight (BW) and gestational age (GA) (BW/GA) > P90)), prematurity (GA at birth < 37 weeks), shoulder dystocia, hypoglycemia and/or hyperinsulinemia at birth, jaundice, neonatal abnormalities, and stillbirths [3,6,71,72].

For macrosomic and LGA births, the Pedersen hypothesis, adapted by Freinkel, was widely accepted. It suggests that the increase in fetal size could possibly be a result of maternal hyperglycemia. This fact directly influenced the energy and fuel content of the fetus, mediated by the placenta, which may reflect in hyperinsulinemia. The increased availability of glucose and free fatty acids (fuels) via the placenta could stimulate the expression of type 1 insulin-like growth factor (IGF-1), which influences fetal growth, in addition to endogenous fetal insulin production [73,74].

Hyperinsulinemia was suggested to stress the developing pancreatic β-cells, contributing to their dysfunction and insulin resistance, even if still in the uterine environment. This can cause fetal hypoglycemia [3,72]. Additionally, fetal hyperinsulinemia seems to alter the synthesis of pulmonary surfactants, predisposing to respiratory distress syndrome, increasing neonatal morbidity rates [75].

As for prematurity, its risk may be associated with rupture of uterine membranes. In addition, its complications can cause adverse outcomes, such as jaundice, respiratory and feeding difficulties, neonatal morbidity, and mortality, among others [71]. As seen, jaundice can also be secondary to premature birth, but it can also be due to macrosomia. Macrosomic neonates need greater oxygen demand, possibly due to intrauterine fetal hypoxia with increased erythropoiesis and, consequently, polycythemia. When erythrocytes rupture, serum bilirubin concentrations increase, leading to neonatal jaundice [6,71].

Regarding shoulder dystocia, its occurrence has been identified as one of the most severe perinatal complications, dealing with vaginal and birth trauma, with an increased risk of approximately 20 times for brachial plexus injuries [76,77]. It is also noteworthy that the offspring is at potential risk of developing metabolic disorders in the immediate postpartum, probably due to the dependence formed by intrauterine hyperglycemia, which can contribute to brain damage [3,7,78]. Finally, congenital anomalies can be influenced by the maternal hyperglycemic environment, which seems to cause severe damage to the development of fetal organs [71].

Regarding future complications, i.e., in the long term, a recent and innovative line of research has emerged, with a series of studies aimed at investigating the transgenerational relationship between early environment and later adverse outcomes, seeking to understand the potential insights, recognized as fetal and epigenetic programming [68,79]. These lines of research may explain the relationship between the easier development of metabolic disorders (as obesity), during childhood or early adulthood, with children from pregnancy with GDM [80,81,82].

## 4. Inflammatory Aspects in GDM

**Figure 1:**
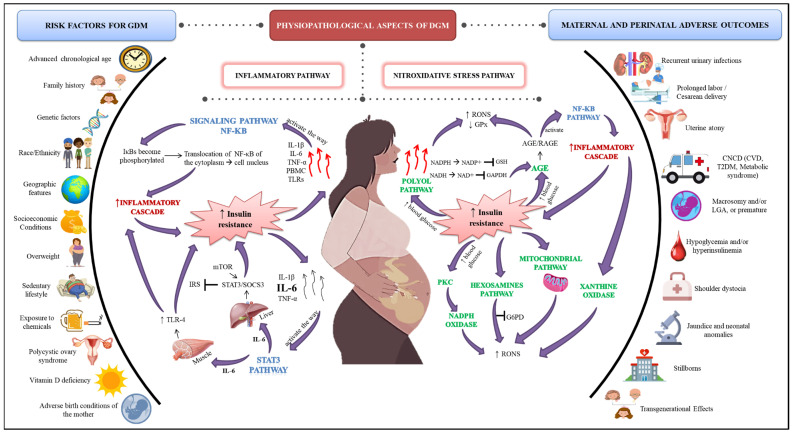
Summarized scheme related to inflammatory and oxidative stress aspects in GDM. GDM: Gestational diabetes mellitus; IL-1β: Interleukin 1β; IL-6: Interleukin 6; TNF-α: Tumor necrosis factor-α; PBMC: Peripheral blood mononuclear cells; TLRs: Toll-like receptor; TLR-4: Toll-like receptor 4; **|─**: Inhibition; IRS: Insulin receptor substrates; mTOR: Mammalian target of rapamycin; RONS: Reactive oxygen and nitrogen species; GPx: Glutathione peroxidase; NADPH: Nicotinamide adenine dinucleotide fosfato; NADP+: n Nicotinamide adenine dinucleotide fosfato oxidized; NADH: Nicotinamide adenine dinucleotide; NAD+: Nicotinamide adenine dinucleotide oxidized; G6PD: Glucose-6-phosphate dehydrogenase; AGE: Advanced glycation end products; RAGE: Receptor of AGE; CNCD: Chronic non-communicable diseases; CVD: Cardiovascular diseases; T2DM: Type 2 diabetes mellitus; LGA: Large for gestational age.

As noted so far, the development of GDM involves a range of etiological and pathophysiological factors that are closely related to inflammatory processes (Figure 1). To address the molecular aspects in GDM more deeply, two main inflammatory pathways have been identified: the nuclear factor kappa B (NF-kB) and signal transducers and activators of transcription 3 (STAT3) pathway [83,84]. Importantly, inflammation is secondary to the coordinated activation of multiple signaling pathways. These signaling pathways, after being activated, regulate the expression of pro- and anti-inflammatory mediators [2].

### 4.1. NF-kB Signaling Pathway

NF-kB signaling pathway is the product of interactions between dimeric transcription factors, inhibitory regulators of NF-kB (IκBs), and the IκB kinase complex (IKK), acting in the primary regulation of the inflammatory response, in the control of the innate and adaptive immune system, and in cellular processes, such as differentiation and proliferation [85]. This signaling system is tightly regulated; however, its dysregulation has implications for inflammatory and pathological processes, depending on the increased expression of pro-inflammatory agents, including cytokines, chemokines and adhesion molecules [86].

In recent years, two distinct paths have been proposed for the activation of the NF-kB pathway, called the “canonical” and “alternative” pathway. These pathways are differentiated by the IKK complex, which is divided into three subunits, two kinases (IKKα (necessary for the activation of the alternative pathway through phosphorylation and processing of p100, the precursor of p52, independent of IKKβ and IKKγ), and IKKβ (fundamental in the activation of the canonical pathway, through the phosphorylation of IκBs, which requires the subunit IKKγ, but not IKKα)) and a regulator (IKKγ (NEMO)—indispensable in the canonical way) [87].

The canonical pathway is activated by toll-like receptors (TLRs) and pro-inflammatory cytokines (interleukin 1β (IL-1β), interleukin 6 (IL-6), and tumor necrosis factor α (TNF-α)), leading to the activation of NF-kB dimers; RelA or cRel; and with this, the regulation of the expression of pro-inflammatory genes and cell survival. The "alternative" pathway is activated by CD40 ligand, cytokines of the lymphotoxin family (TNF-β), and B cell activating factor (BAFF) but not by TNF-α, resulting in the complex activation RelB/p52 [85,87].

A wide spectrum of stimuli activated the NF-kB pathway. The IκBs proteins become phosphorylated by the IκB-kinaseβ (IKBKβ), and then, they are degraded by proteasome complexes, allowing for the translocation of NF-κB proteins from the cytoplasm to the nucleus. This induces the activation of the genetic transcription of numerous molecules that initiate the inflammatory cascade involved in various pathologies, such as those arising from insulin resistance (metabolic syndrome, obesity, and diabetes mellitus, among others) [86].

Studies have shown that individuals with insulin resistance had alterations in the expression of peripheral blood mononuclear cells (PBMC), which are involved in NF-kB signaling pathways, and may demonstrate the relationship between insulin resistance and low-grade inflammation [86,88,89]. Based on this assumption, and since the GDM is the result of a transitory process composed of the interaction of insulin resistance and pro-inflammatory mediators, this relationship can be observed from two perspectives, as a potential influencer or product of the activation of the NF-kB pathway.

As an influencer, the GDM can act in the following ways: (1) The pathophysiological processes and etiological factors of GDM involve the increase of pro-inflammatory cytokines, and these cytokines can activate the NF-kB signaling pathway. (2) Insulin resistance can alter the expression of PBMC, and this reflects the activation of the NF-kB pathway. (3) Insulin resistance, followed by hyperglycemia, has the capacity to increase the expression of TLRs subtypes and, thus, to activate the canonical NF-kB signaling pathway [2,83,86,90,91]. All of these pathways can activate the NF-kB pathway and, therefore, can intensify and expand the underlying inflammatory processes, which can negatively impact the health of the maternal–fetal binomial, in the short and long terms [3,72,92].

On the other hand, as a product of the NF-Kb pathway, GDM can occur from failures in the metabolic repair processes, since the pregnancy period, independently, presents inflammatory aspects and oxidative stress in addition to making up a state of insulin resistance due to the priority supply of glucose to the fetus and placental tissues. In this context, in situations where there are defects in the inhibition or suppression of the NF-kB pathway, the inflammatory cascade can exacerbate the underlying inflammation from the increased expression of pro-inflammatory cytokines, intensifying insulin resistance, with subsequent hyperglycemia and, with this, acting in the development of GDM, which can also cause the occurrence of maternal and perinatal outcomes [3,72,92].

### 4.2. STAT3 Signaling Pathway

Signal transducers and activators of transcription (STAT) represent a family of transcription factors mediated by the Janus kinase pathway (JAK)/STAT [93]. STAT regulates the expression of genes involved in several essential biological functions (aspects related to cell growth, proliferation, differentiation, signaling, development, and survival, among others) in response to adipokines, proinflammatory cytokines, and growth factors [94,95].

It is noteworthy that the JAK/STAT pathway has strict regulations to maintain physiologically normal conditions. However, studies have observed that derangements or disturbances in the regulation of STAT factors may be associated with pathological processes [84,94,96,97]. The erroneous modulation of this pathway can lead to an activation of STAT signaling, which induces an uncontrolled inflammatory and/or immune response [95]. It is noteworthy that the STAT signaling pathway can be activated by other pathways in addition to JAK/STAT, with the same disorderly impact on the inflammatory and immune response.

Currently, in mammals, seven distinct proteins are known in the STAT family, the STAT 1, 2, 3, 4, 5a, 5b, and 6, each of them encoded by a different gene and subjected to alternative RNA splicing (i.e., removing introns from the precursor ribonucleic acid (RNA), producing a functional mature mRNA), or post-translation proteolytic processing [93,94]. Each member of the STAT family plays a unique role in signal transduction and is crucial to mediating cellular responses to different types of cytokines [93].

Among these members, STAT3 has stood out, mainly for its involvement with insulin resistance and related pathologies, including GDM [2,3,84,97]. STAT3, after nuclear stimulation and translocation, can be expressed by various metabolic tissues, being activated through phosphorylation of tyrosine 705 (Tyr705) and tyrosine 727 (Tyr727), in response to the expression of adipokines, proinflammatory cytokines, and growth factors [98]. In parallel with the activation of the STAT3 pathway, others are also activated, such as that of the suppressor of cytokine signaling 3 (SOSC3), a potential negative feedback inhibitor of the action of proinflammatory cytokines, which can have pro- and anti-inflammatory effects [99].

Several studies have investigated the relationship between STAT3 activation and insulin resistance, indicating mediation between specific tissues (hepatic and muscle), cytokines (IL-6), and adipokines (visfatin), in addition to the signpost SOSC3/STAT3 [96,97,98,100,101,102]. It is noteworthy that IL-6 has been considered a mediator between inflammatory processes derived from insulin resistance conditions. Visfatin, an adipokine present mainly in visceral adipose tissue, which induces the production of IL-6, IL-1β, and TNF-α, has a crucial inflammatory impact on the liver and maybe also related to insulin resistance [96,100,103,104].

The mechanism of IL-6-induced insulin resistance in the liver involves the activation of STAT3 and subsequent induction of the SOCS3 suppressor. SOCS3 inhibits insulin signaling through several distinct mechanisms, including insulin receptor inhibition (IRS), blocking its activation and inducing its degradation [96]. Overexpression of SOCS3 in liver tissues induces insulin resistance [96,100]. These IL-6-induced processes can occur through a transmembrane complex, by activation of the JAK pathway, and consequently of STAT3, dependent in addition to Tyr705 and Tyr727, of Serine 727 (Ser727) [93,94,95].

The phosphorylation of STAT3 through Ser727 amplifies its activity. Therefore, this phosphorylation from Ser727 may be due to specific protein kinases being determined according to the cellular context, including the rapamycin target in mammals (mammalian target of rapamycin—mTOR) [96,105]. The participation of mTOR in the SOSC3/STAT3 pathway, induced by IL-6, can intensify insulin resistance, causing the development of related pathological disorders, such as T2DM and GDM [2,3,84,97].

In muscles, the mechanisms are similar to those in the liver, mediated by the expression of IL-6, which may be elevated in obese and/or insulin-resistant individuals [100]. A parenthesis must be opened at this time; as seen in the previous topic, IL-6 also participates in the canonical signaling pathway of NF-kB and can be activated from TLRs [85,87]. Evidences indicate that insulin resistance can impact the increase of IL-6, and in TLRs, more specifically, type 4 (TLR-4), these mechanisms aremediated by the STAT3 pathway in skeletal muscle [100,106].

These mechanisms involving the STAT3 pathway may participate in the physiopathogenesis of GDM and may be induced by insulin resistance. This culminates in the increased expression of pro-inflammatory cytokines and adipokines, leading activate mechanisms that mediate STAT3 activation, contributing to the underlying systemic inflammation. However, as this is the gestational period, the findings are limited since numerous metabolic interactions can occur, either to contribute to or induce the processes. Among the important factors, there are those intrinsic to pregnancy, the placenta, diabetogenic hormones, in addition to the specific etiological factors in each pregnancy. Additionally, we can observe interway communication, which can simultaneously amplify and intensify inflammation, reflecting on the increased risk for the development of GDM as well as adverse maternal and perinatal outcomes in the short and long terms [2,3,84,97].

## 5. Nitroxidative Stress in GDM

Oxidative stress is the imbalance between cellular oxidants and antioxidants in favor of oxidants, leading to a disruption in redox signaling and/or molecular damage. The products of oxygen reduction are called reactive oxygen species (ROS). In addition to ROS, reactive nitrogen species (RNS) also have a notable impact on redox biology, and as a consequence, on redox imbalance [107,108,109]. It is important to highlight that RONS play an important role in organic homeostasis, being essential as cell signaling molecules.

To maintain homeostasis, the antioxidant defense system acts to neutralize the potentially harmful effects of RONS. In the first line of defense, enzymatic antioxidants come into play, including superoxide dismutase (SOD); catalase (CAT); and the phase 2 reactions, which include the glutathione family, such as glutathione peroxidase (GPx), glutathione reductase (GR), and glutathione S-transferase (GST). In addition to these, non-enzymatic antioxidants also act directly on reactive species or serve as a substrate for the synthesis of enzymatic antioxidants, especially the reduced glutathione (GSH), vitamins C and E, selenium, zinc, manganese, and copper [110,111].

The term oxidative stress biomarkers was created to classify molecules that are modifiable through interaction with RONs as well as with the antioxidant defense system, which changes in response to redox imbalance, that is, they are potentially relevant in the assessment of disease state also in the appreciation of antioxidant effects to optimize health states. Among these biomarkers, the most promising are those closely correlated with the pathophysiological process of the disease [112].

During the gestational period, there is a process of oxidative stress considered low-grade or physiological, since pregnancy has a high demand for oxygen from the progenitress, fetus, and placenta. The placenta, in turn, is an organ rich in mitochondria, resulting in a greater production of RONS [113,114]. Despite its important physiological role, especially during pregnancy, the excessive production of RONS can override the antioxidant defense system, contributing to oxidative damage and thus causing considerable damage to women with GDM, from cell injury to death [115,116].

The oxidative stress present in GDM, well documented in the literature, is due to hyperglycemia and can promote early embryonic development, leading to changes in the main transcription factors and, thus, modifying gene expression [117]. Additionally, increased oxidative stress and inflammation, which arise as a consequence of redox imbalance, can further aggravate insulin resistance in GDM and promote deoxyribonucleic acid (DNA) damage and chromosomal aberrations [118].

The metabolic pathways through which GDM hyperglycemia is able to induce oxidative stress as well as cell and tissue damage are polyol pathway, formation of advanced glycation end products (AGE), activation of protein kinase C (PKC), hexosamine pathway, nicotinamide adenine dinucleotide phosphate (NADPH) oxidase, xanthine oxidase, and mitochondrial pathway (Figure 1) [1], which is described below.

### 5.1. Polyol Pathway

The polyol pathway is responsible for converting glucose to fructose, and this process initially includes the reduction of glucose to sorbitol, which is oxidized to fructose. The aldose reductase enzyme is part of this conversion and has a low affinity for glucose. Thus, in normoglycemic states, glucose metabolism through this pathway is insignificant. However, in a hyperglycemic environment, as in GDM, hexokinase, becomes saturated and excess glucose enters the polyol pathway, where aldose reductase reduces this to sorbitol, while NADPH is oxidized to nicotinamide adenine dinucleotide phosphate oxidized (NADP^+^). Then, sorbitol dehydrogenase can oxidize sorbitol to fructose, producing nicotinamide adenine dinucleotide (NADH) from NAD^+^ (its oxidized form). However, in GDM, where there are high levels of blood glucose, the reaction changes to the generation of sorbitol, and the high consumption of NADPH by this pathway inhibits the replacement of GSH, necessary for the maintenance of GPx activity, culminating in reduced cellular antioxidant response [1,119,120].

One of the mechanisms proposed for the deleterious effects of hyperglycemia via polyol occurs in the cytosol, which includes a decrease in NADPH, thus diminishing the NADPH/NAD^+^ ratio and consequently decreasing the activity of glyceraldehyde-3-phosphate dehydrogenase (GAPDH, a key glycolytic enzyme), causing all glycolytic intermediates that are upstream from GAPDH to increase. Among these, glyceraldehyde-3-phosphate activates the pathway of formation of advanced glycation end-products (AGEs), since its main intracellular precursor (methylglyoxal) is formed from glyceraldehyde-3-phosphate [119,120].

However, an inadequate cytosolic ratio of NADPH to NAD^+^ inhibits the activity of GAPDH, causing accumulation of intracellular glucose, thus promoting the increase in available substrate for complex I of the mitochondrial respiratory chain, as the latter is an important source of ROS in the GDM. The transfer of additional electrons can further increase mitochondrial ROS production via the polyol pathway, and in a cyclic process, high concentrations of ROS also inhibit GAPDH activity. Furthermore, the decreases of GAPDH results from the accumulation of metabolites as fructose and triose phosphates, giving rise to highly reactive dicarbonyls derivatives such as glyoxal and methylglyoxal, and 3-deoxyglucose, which can lead to covalent modification of proteins, giving rise to AGEs. Therefore, polyol leads to decreased NADPH, GSH, and antioxidants, favoring higher production of ROS in the intracellular environment [119,120].

### 5.2. AGEs

It consists of a heterogeneous group of molecules arising from the non-enzymatic reaction between reducing sugars and amino groups of lipids, proteins, and DNA. Despite the formation of these compounds in normal physiological states, the hyperglycemic state and oxidative stress favor their appearance in a more pronounced way [121].

In GDM, the reaction between glucose and proteins leads to Amadori products, a stable ketoamine. This is generated from the interaction between glucose aldehyde groups and free amino groups in proteins, giving rise to Schiff bases, which stabilize, giving rise to Amadori products. These are degraded into other reactive compounds, such as methylglyoxal, capable of reacting directly with amino groups of proteins, thus forming the AGEs [1,120].

It is important to emphasize that such reactions can impair protein function and that diabetic individual contains more significant amounts of plasma and tissue AGEs. Therefore, in this clinical condition, the formation of AGEs is considered an essential source of free radicals, promoting oxidative stress and inflammation. Furthermore, a persistent hyperglycemic state during pregnancy is one of the main factors for abnormal fetal development [9,122].

In addition, extracellular AGEs bind to their receptor (RAGE), which is able to activate transcription factors such as NF-κB and stimulate ROS formation through NADPH oxidase, and this, consequently, can lead to cell injury and damage. This process occurs more exacerbated during pregnancy, and the AGE-RAGE complex plays a fundamental role in regulating pro-inflammatory mediators, such as cytokines, and endothelial dysfunction, via NF-κB [120].

### 5.3. PKC

It consists of a family of kinases composed of different isoforms, differing activation sites. When activated, they induce biological processes such as cell proliferation and differentiation, transmembrane ion transport, glucose metabolism, and others. Among the functions of PKC, there is the activation of mitochondrial NADPH oxidase, resulting in increased oxidative stress. Furthermore, activation of NADPH reduces GSH levels, impairing the antioxidant defense system. Hyperglycemic states can activate PKC isoforms, favoring increased ROS production through NADPH oxidase [119,123].

### 5.4. Hexosamine Pathway

The hexosamine biosynthetic pathway is inherent to glucose metabolism; however, its activation is more pronounced in a hyperglycemic state. This pathway uses glycolysis-derived fructose-6-phosphate to metabolize glucosamine-6-phosphate, an inhibitor of the enzyme glucose-6-phosphate dehydrogenase (G6PD)—the G6PD acts as a pentose phosphate pathway limiter. In turn, the last pathway is an alternative for glycolysis and responsible for the cellular production of NADPH, with this one being utilized in maintaining the redox state by reducing GSSG to GSH [1,120].

Therefore, the inhibition of G6PD through glucosamine-6-phosphate leads to lower NADPH levels, reduced GSH, and, consequently, increased oxidative stress. Furthermore, under hyperglycemic conditions, as in GDM, the hexosamine pathway can stimulate post-translational changes in proteins through glycosylation and synthesis of glycolipids, proteoglycans, and glycosylphosphatidylinositol anchors. Thus, this pathway can lead to pathological changes in gene expression, which may be associated with persistent hyperglycemia and complications of the disease [119].

### 5.5. NADPH Oxidase

It consists of an enzyme complex located in the cytosol in which the main role is the production of ROS, through the transport of electrons, and is therefore essential in redox signaling. In hyperglycemia, it can be stimulated by AGEs, insulin and angiotensin II. Furthermore, hypoxia is also capable of inducing such stimuli to NADPH oxidase. When activated by hyperglycemia, NADPH oxidase catalyzes the transfer of electrons for the production of superoxide radical anion (O_2_^•−^) and hydrogen peroxide (H_2_O_2_) from molecular oxygen, i.e., high levels of glucose increase the production of ROS through NADPH oxidase [1,108].

Data from the literature in animals show greater expression and activation of NADPH oxidase in endothelial and placental cells in the presence of GDM as well as suggest that this pathway is the main enzymatic source of O_2_^•−^ in the placenta [124].

### 5.6. Xanthine Oxidase

Xanthine oxidase is an enzyme responsible for catalyzing the oxidation of xanthine to uric acid and is considered an essential source of O_2_^•−^. Its activity and plasma levels can be increased in the presence of inflammatory agents, especially interferon. However, its most relevant role is related to hypoxia/reperfusion processes [108,121].

During this process, a cytosolic calcium increase may occur, favoring the activation of the protease calpain, which is responsible for promoting the breakdown of a peptide bridge of the xanthine dehydrogenase, which culminates in the formation of xanthine oxidase. In turn, xanthine oxidase requires oxygen to promote the conversion of hypoxanthine to xanthine. Thus, during ischemia, these two substances accumulate. When reperfusion occurs, hypoxanthine is oxidized to xanthine and, afterward, to uric acid, causing the formation of O_2_^•−^ and H_2_O_2_ and, in the presence of transition metals, the formation of ^•^OH. Due to the activation of this pathway and the increase in intracellular calcium, non-specific proteases and phospholipases are activated and result in the synthesis of inflammatory mediators, including prostaglandins, leukotrienes, and thromboxanes [108,110].

As mentioned earlier, it is known in the redox area that inflammation/hypoxia-induced elevation of xanthine oxidase activity leads to increased ROS generation and consequently to adverse human health outcomes. However, a recent and provocative discovery has been identified, particularly under conditions of hypoxia or anoxia, where xanthine oxidase catalyzes the reduction of NO_2_^−^ to ^•^NO, from reducing substrates such as NADPH, with beneficial effect. In turn, ^•^NO can react with ROS, which were previously produced by xanthine oxidase, as O_2_^•−^, leading to the formation of peroxynitrite (ONOO), a powerful oxidizing agent. Therefore, in GDM, the xanthine oxidase pathway is an important source of RONS [108].

### 5.7. Mitochondrial Pathway

Mitochondria are naturally the primary cellular sources of ROS, mainly produced during oxidative phosphorylation due to the leakage of electrons from the electron transport chain located in the mitochondrial membrane. Once formed, ROS can favor mitochondrial damage or play an important role in redox signaling from mitochondria to other cell compartments. The primary process of ROS elimination in mitochondria occurs through the presence of O_2_^•−^, which does not cross the mitochondrial membrane. Manganese superoxide dismutase (MnSOD) converts this into H_2_O_2_, which is later converted into water by GPx [1,108,119].

It is noteworthy that, during a healthy pregnancy, there is an increase in mitochondrial activity, particularly via the placenta (complex and membranous vascular organ, specific in the gestational period, capable of promoting the metabolic interaction between mother and fetus, through the transport of nutrients and oxygen, elimination of fetal metabolites, and hormonal production), which can lead to greater formation of ROS, greater generation of O_2_^•−^ from NADPH oxidase, and alteration in the organic capacity to remove such compounds [125,126,127,128,129,130,131,132].

In turn, in cells with a high concentration of glucose, as in GDM, there is a more significant amount of glucose derived from pyruvate, with the latter being oxidized to the tricarboxylic acid (TCA), a product of the metabolism of glycolysis capable of initiating the glycation of intracellular proteins, which leads to an increase in the flow of electron donors, specifically NADH and reduced flavin adenine dinucleotide (FADH2), in the electron transport chain. Consequently, the voltage gradient of the mitochondrial membrane increases to a limit considered critical. Thus, within the mitochondrial electron transport chain, electrons leak, which is transferred to molecular oxygen, generating O_2_^•−^. Furthermore, the increase in ROS resulting from hyperglycemia can promote morphological changes in mitochondria [1,119].

In summary, the high concentrations of glucose in muscle, fat, and pancreatic cells that occur in the GDM lead to increased production of ROS, which despite playing an essential role, especially as signaling molecules in the body, in excess, induces mitochondrial dysfunction and reduced production of adenosine triphosphate (ATP). Thus, it consequently decreases the activity of GAPDH, increasing the flow of the polyol pathway, stimulates PKC, increases the production of AGEs within cells, and excites the hexosamine pathway, establishing a close relationship between hyperglycemia and oxidative stress [108,119]. Therefore, a hyperglycemic cellular environment is associated with oxidative stress, characterized by the overproduction of free radicals and reduced ability to remove RONS by the antioxidant defense system, which culminates in the appearance of clinical signs and symptoms of the disease [1].

## 6. Intestinal Microbiota and GDM

The intestinal microbiota refers to all microorganisms that colonize the human gastrointestinal tract. Resident microorganisms have a symbiotic relationship with the host. They can extract energy from molecules that humans cannot digest, producing bioactive compounds and SCFA, which lead to several benefits to host metabolism. Therefore, the microbiota is currently considered an endocrine-metabolic organ, capable of controlling various organic processes [17,132,133].

**Figure 2:**
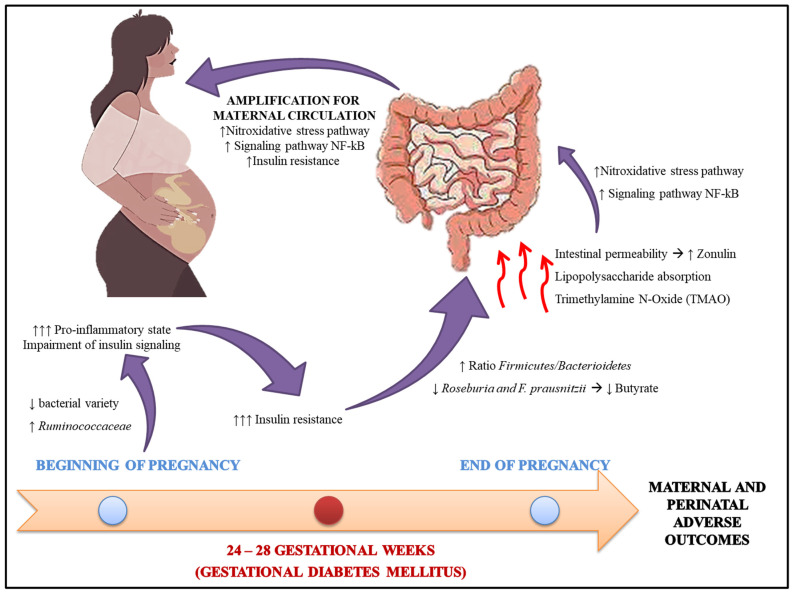
Scheme of the interaction between GDM and intestinal microbiota, inflammatory, and oxidative stress processes.

In turn, the change in the microbiota composition is called dysbiosis. This condition plays a crucial role in several pathogenic processes of metabolic diseases, such as obesity and diabetes mellitus. Among the mechanisms through which dysbiosis can compromise metabolism, there is an increase in intestinal permeability, increased LPS absorption, abnormal SCFA production, altered conversion of primary to secondary bile acids, and increased bacterial production of toxic substances such as trimethylamine N-oxide (TMAO) [134,135]. Thus, such changes lead to activating inflammatory processes and autoimmune pathways, autoantigen mimics, impaired insulin signaling, and others (Figure 2) [136].

Several factors can influence the composition of the microbiota, including early life events (genetic factors, premature birth, and breastfeeding), as well as future events (presence of comorbidities, diet composition, use of prebiotics and probiotics, use of antibiotics, and pregnancy) [3,17]. In a healthy pregnancy, the microbiota undergoes several changes between the gestational trimesters. Studies show that healthy women at the end of pregnancy presented a microbiota composition similar to non-pregnant individuals with metabolic syndrome [3,137].

The complex hormonal, immunological, and metabolic changes in the maternal organism promote maternal weight gain, increased concentrations of pro-inflammatory cytokines, and insulin resistance. However, reducing insulin sensitivity in healthy pregnancies is beneficial as it aims to promote fetal growth and to increase nutrient absorption [3]. In GDM, marked insulin resistance promotes glucose intolerance. In general, insulin resistance is associated with a higher firmicutes/bacterioidetes ratio and a reduction in the amount of butyrate (an SCFA) producing bacteria, such as Roseburia and Faecalibacterium prausnitzii [138,139]. However, it is unclear whether the altered microbiota is a cause or consequence of GDM [17].

Data from the literature indicate a different composition of the microbiota in early pregnancy, before the development of GDM, since both conditions reduce the variety of bacteria and increase the Ruminococcaceae family, with a higher pro-inflammatory state and impaired insulin signaling [3,140]. Furthermore, in GDM, intestinal permeability may improve, which is regulated by junction proteins such as zonulin (ZO-1); when it is accessible in plasma, it is associated with GDM [141]. This fact can favor the movement of inflammatory mediators from the intestine to the circulation, promoting even more insulin resistance [3,142].

A study conducted in women with GDM to assess the composition of the intestinal, oral, and vaginal microbiota, and its relationship with the disease, found a specific composition of the intestinal and vaginal microbiota, less diverse than the control group, suggestive of dysbiosis and indicating the involvement of these changes with the GDM [143]. Corroborating this finding, through analyzes of the microbiota of the maternal (oral, intestinal and vaginal) and child (oral, pharyngeal, meconium and amniotic fluid) pairs, another study identified changes in the microbiota of the pairs belonging to the group with GDM, when compared with the control, namely lesser diversity and greater abundance of some viruses (herpesviruses and mastadenoviruses, for example) [15]. Furthermore, the same trend was observed in maternal and neonatal changes in the GDM, reinforcing the intergenerational microbiotic agreement associated with the disease [15].

It is essential to highlight that the microbiota of women with GDM can be transmitted to their fetuses. Thus, the knowledge of the composition and early microbiota modulation is exceptionally notorious. However, the link between dysbiosis, GDM, and inflammation has not been fully elucidated due to the scarcity of scientific studies [15,17,144].

Considering that women who had GDM are at higher risk of having it again in subsequent pregnancies and T2DM, prevention strategies should be adopted, such as lifestyle modifications, including exercise and dietary changes, to better health outcomes. In addition, women with GDM who adopted dietary recommendations had reduced Bacteroides and better glycemic control [145].

Furthermore, an alternative to be considered is the modulation of the microbiota in the GDM. Probiotics are microorganisms that promote health benefits to the host [146]. Bifidobacterium and Lactobacillus are the most widely used for this purpose [147]. This procedure can promote the better composition of the intestinal microbiota; reduce the adherence of pathobionts; strengthen intestinal permeability; aid the immune response, insulin signaling, and energy metabolism; be a safe alternative, is well-tolerated, and has proven beneficial effects in various clinical conditions, including GDM. However, few clinical studies with probiotics are available in the literature in pregnant women, especially in GDM [144]. Table 1 provides a qualitative summary of the clinical trials, which performed probiotic supplementation, alone or in combination, for the treatment of GDM.

Regarding the action of probiotics on inflammation in GDM, the literature is scarce. However, increasing evidence has shown beneficial effects of probiotic supplementation on intestinal health, from the attenuation of inflammatory processes and oxidative stress, by mechanisms that involve the inhibition of the NF-κB pathway, being characterized as a well-documented change in the GDM [148,149]. Interestingly, probiotics exert acute biological effects, highlighting their antioxidant role, which remains controversial [150]. In this sense, a study conducted in an animal model promoted probiotic supplementation in rats with GDM for 18 days. Serum levels of malondialdehyde (MDA), SOD, GR, and GPx showed that the antioxidant mixture reduced the induced oxidative stress [151].

In addition to probiotics, intestinal modulation includes other factors, such as diet, capable of influencing the composition of the microbiota directly and indirectly. Some nutrients can directly interact with the microbiome and can stimulate the host’s metabolism and immune system, thus promoting changes in the microbiota [17]. Few studies have evaluated the role of maternal nutrition on the microbiota during pregnancy. In general, high fiber consumption is associated with greater bacterial richness. On the other hand, low fiber consumption, associated with high consumption of fat, especially saturated, favors lower microbiota richness [17].

In this context, a study observed the impact of diet on the intestinal microbiota in GDM. Women aged 24–28 weeks who received dietary recommendations and experienced up to 38 weeks of gestation were included. There was a significant reduction in the adherence of Bacteroides, which is associated with diets rich in animal fat. In addition, at baseline, total fat intake was associated with higher amounts of Alistipes and protein intake with *Faecalibacterium*. On the other hand, at the end of the research, fiber consumption was associated with the genus *Roseburia*. However, none of these bacteria were associated with the metabolic changes that occur in the GDM [145].

Still, a study that evaluated fecal bacteria from women who had previous GDM reported a lower proportion of the *Firmicutes phylum* and a more significant proportion of the Prevotellaceae family, compared with those with normoglycemia [152]. Firmicutes metabolize dietary plant polysaccharides, which increase their levels. In turn, the consumption of animal protein and red meat promotes the intestinal reduction in *Firmicutes*. Therefore, these bacteria seem to be relevant in the pathogenesis of GDM, regardless of diet, by still unknown mechanisms [3]. Given the above, the need for further studies on the role of the microbiota in GDM is evident and the promising beneficial effects that probiotics can bring in this condition. Thus, the conduction of clinical trials of modulation of the microbiota and, with dietary manipulation strategies in the GDM, are significant to assess the possible use of these for the prevention and control of the disease. Furthermore, microbiota modulation is a potential therapy for GDM [17].

**Table 1:** Randomized clinical trials with supplementation of probiotics, alone or in combination, for the treatment of gestational diabetes mellitus.

Source Sample	Population	Size *	Supplementation	Dose/Duration	Main Findings
Karamali et al. (2016) [118]	Iran	I: 30 C: 30	*L. acidophilus + L. casei + B. bifidum*	2 × 10^9^ CFU/ 6 weeks	Supplementation with probiotics ↓FBG, serum insulin, TG, and VLDL-c, and improved insulin resistance indexes.
Hajifaraji et al. (2018) [150]	Iran	I: 27 C: 29	*L. acidophilus LA-5 + B. BB-12 + S. thermophilus STY-31 + L. delbrueckii bulgaricus + LBY-27*	>4 × 10^9^ CFU/ 8 weeks	Supplementation with probiotics significantly ↓CRP and TNF-α. MDA, GPx and GR in women in the intervention group.
Kijmanawat et al. (2019) [153]	Thailand	I: 28 C: 29	*Bifidobacterium + Lactobacillus*	2 × 10^9^ CFU/ 4 weeks	In women with diet-controlled GDM, supplementation with probiotics ↓FBG and insulin resistance compared with the control.
Babadi et al. (2018) [154]	Iran	I: 24 C: 24	*L. casei + B. bifidum + L. fermentum + L. acidophilus*	2 × 10^9^ CFU/ 6 weeks	Probiotic supplementation improved the expression of genes related to insulin; glycemic control; inflammation; lipid profile, and oxidative stress markers, such as ↓MDA and ↑TAC, compared with the control.
Badehnoosh et al. (2018) [155]	Iran	I: 30 C: 30	*L. acidophilus + L. casei + B. bifidum*	2 × 10^9^ CFU/ 6 weeks	Probiotic supplementation improved FBG, and CRP, ↑TAC, and ↓MDA, without affecting pregnancy outcomes.
Nabhani et al. (2018) [156]	Iran	I: 45 C: 45	*L. acidophilus + L. plantarum + L. fermentum + L. gasseri +* FOS	1.5–7.0 × 10^9–10^ CFU + 38.5 mg/ 6 weeks	Symbiotics had no effect on FBG and insulin resistance/sensitivity indexes. However, an ↑ in HDL-c and TAC was seen, and a ↓ was seen in blood pressure in the intervention group.
Jamilian et al. (2019) [157]	Iran	I: 29 C: 28	*L. acidophilus + B. bifidum + L. reuteri + L. fermentum +* Vitamin D	8 × 10^9^ CFU/ 6 weeks +50.000 UI every 2 weeks	↓FBG, serum insulin, CRP, and MDA; ↑TAC and GSH; and improved insulin resistance scores.
Karamali et al. (2018) [158]	Iran	I: 30 C: 30	*L. acidophilus + L. casei + B. bifidum* + Inulin	2 × 10^9^ CFU/ 6 weeks +800 mg	Symbiotic supplementation ↓CRP and MDA; ↑TAC and GSH; and↓ the rates of cesarean section, hyperbilirubinemia and hospitalization in NB, without affecting other pregnancy outcomes.
Ahmadi et al. (2016) [159]	Iran	I: 35 C: 35	*L. acidophilus + L. casei + B. bifidum* + inulin	2 × 10^9^ CFU/ 6 weeks +800 mg	Symbiotics ↑ insulin metabolism markers, and the insulin sensitivity index as well as ↓VLDL-c and TG.
Jafarnejad et al. (2016) [160]	Iran	I: 41 C: 41	*S. thermophilus + B. breve + B. longum + B. infantis + L. acidophilus + L. plantarum + L. paracasei + L. delbrueckii subsp. Bulgaricus*	15 × 10^9^ CFU/ 8 weeks	No differences were observed in FBG, glycated hemoglobin, serum insulin, and insulin resistance indices. However, ↓CRP, IL-6, and TNF-α were observed, without changes in IL-10 and IFN-γ.
Dolatkhah et al. (2015) [161]	Turkey	I: 29 C: 27	*L. acidophilus LA-5 + B. BB-12 + Streptococcus thermophilus + STY-31 + L. delbrueckii bulgaricus LBY-27*	>4 × 10^9^ CFU/ 8 weeks	↓FBG and insulin resistance index, and less weight gain in those in the intervention group.
Lindsay et al. (2015) [162]	Ireland	I: 74 C: 75	*L. salivarius*	1 × 10^9^ CFU/ 6 weeks	No beneficial effect on glycemic control or pregnancy outcomes. ↓ in total and LDL-c in the supplemented group.

* Pregnant with GDM; I: Intervention; C: Control; GDM: Gestational diabetes mellitus; ↑: Increase; ↓: Decrease; B: Bifidobacterium; FBG: Fasting blood glucose; FOS: Fructooligosaccharide; TG: Triglycerides; CRP: C-reactive protein; TNF-α: Tumor necrosis factor α; GPx: Glutathione peroxidase; GR: Glutathione reductase; GSH: Glutathione; HDL-c: High-density lipoprotein cholesterol; IFN-γ: Interferon gama; IL-6: Interleukin 6; IL-10: Interleukin 10; L: Lactobacillus; LDL-c: Low-density lipoprotein cholesterol; MDA: Malondialdehyde; TAC: Total antioxidant capacity; NB: Newborns; CFU: Colony forming unit; VLDL-c: Very low-density lipoprotein cholesterol.

## 7. Alternative Therapies in GDM

Currently, the first line of care recognized in the treatment of GDM refers to changes in lifestyle, including dietary changes and abandoning a sedentary lifestyle. Concomitant with this strategy, the use of insulin and/or oral antidiabetics has been recommended [163]. However, the pathophysiological process of GDM also involves the participation of pro-inflammatory cytokines (IL-1β, IL-6, and TNF-α, among others) and oxidative stress (increase in RONS and AGEs, and decrease in antioxidant agents, among others) which, when associated, favor the exacerbation of insulin resistance, associated with hyperglycemia [8,13].

Current drug therapies aim to attenuate insulin resistance and control maternal blood glucose. Therefore, if insulin resistance is not treated adequately, that is, with the introduction of agents with antioxidant and anti-inflammatory properties, performing a combination therapy with insulin and/or oral antidiabetics, it may maintain the vicious cycle of insulin resistance, which is followed by maternal hyperglycemia and failure to attenuate adverse maternal and perinatal outcomes [6,8].

This context motivated the investigation of several therapeutic agents on GDM, which could alleviate the symptoms and improve the plasma expression of not only glucose but also insulin, and underlying inflammatory and oxidative factors. In this sense, the scientific literature has extensively reports on such therapies, whether in human beings or animal models. Thus, we separated this topic into two subsections, Randomized Clinical Trials and Experimental Studies in Animal Models.

### 7.1. Randomized Clinical Trials

Table 2 lists randomized clinical trials that analyzed alternative approaches that complemented conventional medicine, based on natural products, diets, and nutrients, with active principles capable of attenuating the pathophysiological signs and symptoms of the underlying disease as well as the future repercussions caused by them to the maternal-fetal binomial [164].

Among these alternative approaches, investigations identified that some of them would have a positive influence only on the attenuation of inflammatory processes and oxidative stress, such as selenium [165], olive oil [166], fish oil [167], and soy oligosaccharide [168], and others only on optimizing insulin sensitivity or by decreasing insulin resistance, such as pepper with capsaicin [169]; mugwort [170]; the dietary approaches to stop hypertension (DASH) diet [171]; co-supplementation of magnesium, zinc, calcium, and vitamin D [172]; vitamin D supplementation associated with evening primrose oil [173] and ginger [174]. Co-supplementation of vitamin D and calcium [175,176], in addition to co-supplementation of zinc and vitamin E [177], cod liver oil [178], flaxseed oil [179], and selenium [180] participate positively to reduce inflammatory processes, oxidative stress, and insulin resistance, contributing favorably to normal serum glucose levels.

Natural oils (olive oil, fish oil, cod liver, and flaxseed) are sources of monounsaturated fatty acids (MUFA (olive oil)) and polyunsaturated ones (PUFAS (fish oil, cod liver, and flaxseed)). MUFAs are endogenous ligands of peroxisome proliferator-activated receptors (PPARs—transcription factors activated by ligands capable of regulating metabolic and anti-inflammatory pathways). In women with GDM, the expression of PPARs is reduced, so the use of ligands such as MUFAs can increase their serum levels and thus contribute to the elevation of anti-inflammatory factors, thus attenuating the underlying inflammation in the GDM. Additionally, it is noteworthy that the expression of PPARs may involve epigenetic mechanisms since this transcription factor is the target of some microRNAs. Thus, a possible future research perspective deals with these factors’ dietary modulation [166].

The PUFAS, especially those of the omega-3 family (ω-3), such as α-linolenic acid (ALA), eicosapentaenoic acid (EPA), and docosahexaenoic acid (DHA), can also be associated with anti-inflammatory and antioxidant mechanisms in GDM by inhibiting the phosphatidylinositol-3-kinase (PI3K) and protein kinase B (PKB) pathways, thus attenuating the expression of NF-kB, which reflects the decrease in the manifestation of pro-inflammatory cytokines and RONS, improving insulin resistance [167,178,179]. However, despite recognizing some of the anti-inflammatory and antioxidant pathways that these compounds participate in, further studies are still needed. The research needs to focus on establishing a safe daily dose for treatment and prevention and to more deeply identify mechanisms of action from the perspective of nutrigenomics.

While soy oligosaccharides (raffinose, stachyose, and sucrose) are considered potential prebiotics, they can act in the modulation of the intestinal microbiota and, from this, in the prevention and attenuation of various pathological processes, mainly mediated by their antioxidant action, through the increase in the expressions of CAT, SOD, and GPx. However, the related metabolic pathways are not fully elucidated. Researchers believe that this relationship may also exist due to the presence of polyphenolic compounds in soy, which through the reduction of RONS expression, may reflect the systemic relationship between oxidants/antioxidants, improving insulin resistance [168].

Regarding micronutrients, isolated selenium supplementation, as well as co-supplementation of vitamin D + calcium and zinc + vitamin E, also led to the attenuation of the expression of pro-inflammatory cytokines and RONS [165,175,176,177]. Selenium is a trace element that can alleviate the inflammatory response by inhibiting the NF-κβ signaling pathway and TLR through the expression of PPAR-γ, which is a complementary way to attenuate insulin resistance and to improve the performance of glucose transporters (GLUT) and neutralize RONS [165].

Vitamin D and calcium are generally reduced in pregnancies with GDM and are even considered risk factors for developing the disease by the mechanisms observed in Box 3. For this, researchers analyzed vitamin D and calcium supplementation together, as when combined, they can exert activity on oxidative stress through cell cycle regulation, activation of antioxidant enzymes, and PTH suppression, generally improving insulin resistance and the inflammatory processes [175,176]. For zinc associated with vitamin E, its antioxidant and anti-inflammatory effects follow routes similar to those in which selenium participates [177].

**Table 2:** Randomized clinical trials with alternative therapies for the treatment of GDM.

Source	Type of Study	Population	Sample Size *	Intervention	Main Findings
Karamali et al. (2020) [165]	Randomized clinical trial, double blind	Teerã, Iran	I: 18 C: 18	Selenium supplementation (200 μg/day)	↑expression of PPAR-γ and GLUT-1, but did not affect the gene expression of LDLR and LP(a) (*p* < 0.05), compared with the control.
Gomez et al. (2020) [166]	Randomized clinical trial, double blind	Buenos Aires, Argentina	I: 15 C: 15	3 tablespoons of extra virgin olive oil daily	The placenta of pregnant women showed regulation on PPARα expression and pro-inflammatory markers (IL-1β and TNF-α), and ↑the expression of miR-518d (*p* < 0.05), compared with the control.
Gunasegaran et al. (2020) [176]	Randomized clinical trial, double blind	Puducherry, India	I1: 34 I2: 36	I1: Vit D (1000 UI/day) + calcium (1000 mg/day) I2: Vit D (250 UI/day) + calcium (500 mg)	I1↓the serum levels of glucose, insulin, LDL-c, and total cholesterol and ↑the levels of HDL-c and total GSH (*p* < 0.05), compared with I2.
Jamilian et al. (2020) [179]	Randomized clinical trial, double blind	Arak, Iran	I: 30 C: 30	2 flaxseed oil (1000 mg) capsules/day, containing 400 mg of α-linolenic acid	Seriously ↓ levels of glucose, insulin, insulin resistance, VLDL-c, total cholesterol, CRP, and MDA, and ↑ sensitivity to insulin, nitrite, and GSH (*p* < 0.05), compared with the control.
Hajimoosayi et al. (2020) [174]	Randomized clinical trial, double blind	Teerã, Iran	I: 37 C: 33	3 ginger tablets/day (1500 mg), with consumption after main meals	↓serum levels of blood glucose, insulin, and the HOMA-IR index (*p* < 0.05), compared with control.
Yang et al. (2019) [178]	Randomized clinical trial, double blind	Changchun, China	I: 268 C: 270	1 capsule/day of cod liver oil (500 mg)	↓serum levels of glucose, lipids, CRP, HOMA-IR, and adverse perinatal outcomes (*p* < 0.05) compared with the control.
Ostadmohammadi et al. (2019) [177]	Randomized clinical trial, double blind	Kashan, Iran	I: 27 C: 27	Zinc co-supplementation (zinc gluconate 233 mg/day) + Vit E (400 IU/day)	Supplemented group ↓ the serum levels of HOMA-IR insulin, total cholesterol, LDL-c, and QUICKI (*p* < 0.05), compared with the control group. It positively regulated the gene expression of PPAR-γ and LDLR (*p* < 0.05), compared with the control.
Jamilian et al., (2018) [167]	Randomized clinical trial, double blind	Arak, Iran	I: 20 C: 20	2 capsules/day of fish oil (1000 mg), containing 180 mg EPA + 120 mg DHA	↑ expression of PPAR-γ, IL-1, and TNF-α (*p* < 0.05), compared with the control.
Karamali et al. (2018) [172]	Randomized clinical trial, double blind	Teerã, Iran	I: 30 C: 30	Magnesium–zinc–calcium–vitamin D co-supplementation	Co-supplementation: ↓ serum glucose, insulin, HOMA-IR, TG, and VLDL-c, while increasing the QUICKI index (*p* < 0.05) compared with the control.
Gao et al., 2017 [181]	Randomized clinical trial, double blind	Hebei, China	I: 123 C: 121	Enrichment of margarine with phytosterols (every 10 g of margarine, 2 g of phytosterols, twice a day)	Supplementation ↑ maternal and neonatal outcomes in patients with GDM.
Zhang et al., 2017 [182]	Randomized clinical trial, double blind	Shandong, China	I: 176 C: 150	1 capsule/day of EGCG (500 mg)	Supplementation ↑ maternal diabetes parameters and ↓ risk of neonatal complications (*p* < 0.05), compared with the control.
Yuan et al. 2016 [169]	Randomized clinical trial	Chongqing, China	I: 20 C: 22	Pepper with capsaicin (5 mg/day)	Supplementation ↓ serum concentrations of glucose, insulin, HOMA-IR, OGTT, total cholesterol, TG, and the risk for LGA births and ↑ the serum levels of apolipoprotein B and CGRP (*p* < 0.05), compared with the control.
Jamilian et al. 2016 [173]	Randomized clinical trial	Arak, Iran	I: 30 C: 30	1 capsule/day formulated with vit D3 (1000 IU) + evening primrose oil (1000 mg)	Supplementation ↓ serum levels of glucose, insulin, HOMA-IR, QUICKI, VLDL-c, LDL-c, and total cholesterol (*p* < 0.05), compared with the control.
Sun et al. 2016 [170]	Randomized clinical trial	Shandong, China	I: 64 C: 65	2 *Artemisia* extract tablets/day (200 mg)	Supplementation ↑ insulin sensitivity through upregulation of adiponectin.
Asemi et al. 2015 [180]	Randomized clinical trial, double blind	Kashan, Iran	I: 35 C: 35	1 tablet/day of selenium (200 μg)	Significantly ↓ serum glucose, insulin, HOMA-IR, CRP, MDA, and plasma GSH concentrations (*p* < 0.05), compared with the control.
Fei et al. 2014 [168]	Randomized clinical trial	Suzhou, China	I: 46 C: 51	Soy oligosaccharide (10 g/day) in 200–300 mL of warm water, given orally before bedtime	Soybean oligosaccharide: ↑ serum activities of SOD, CAT, and GPx, while ↓ serum levels of TBARS and HOMA-IR (*p* < 0.05), compared with the control.
Asemi et al. 2014 [175]	Randomized clinical trial	Kashan, Iran	I: 25 C: 26	Co-supplementation of calcium (1000 mg/day), and Vit D3 (50,000 U, in two moments, on the 1st and 21st days of the study)	Co-supplemented: significantly ↓ serum concentrations of glucose, insulin, HOMA-IR, LDL-c, and total cholesterol and ↑ in HDL-c levels; it was possible to avoid the increase in MDA, compared with the control.
Asemi et al. 2013 [171]	Randomized clinical trial	Kashan, Iran	I: 16 C: 16	DASH diet (rich in fruits, vegetables, whole grains, and low-fat dairy but low in total fat, saturated, cholesterol, refined grains, and sweets, with a total of 2400 mg/day Na)	The intervention group with DASH diet ↓plasma concentrations of glucose, insulin, and HOMA-IR, (*p* < 0.05), compared with the control.

* Pregnant with GDM; ↑: Increase; ↓: Decrease; I: Intervention; C: Control; EPA: Eicosapentaenoic acid; GDM: Gestational diabetes mellitus; DASH: Dietary approaches to stop hypertension; DHA: Docosahexaenoic acid; PPAR-γ: Peroxisome proliferator; IL-1: Interleukin 1; TNF-α: Tumor necrosis factor α; HOMA-IR: Homeostatic assessment model for insulin resistance; GLUT-1: Glucose transporter 1; LDLR: Low-density lipoprotein receptor; LP(a): Lipoprotein a; CRP: C-reactive protein; MDA: Malonaldehyde; VLDL-c: Very low-density cholesterol; QUICKI: Quantitative Insulin Sensitivity Check Index; HDL-c: High-density cholesterol; OGTT: Oral Glucose Tolerance Test; CGRP: Calcitonin gene-related peptide; EGCG: 3-gallate epigallocatechin; Na: Sodium; LDL-c: Low-density lipoprotein cholesterol; TG: Triglycerides; GSH: Reduced glutathione; LGA: Large for gestational age; SOD: Superoxide dismutase; CAT: Catalase; GPx: Glutathione peroxidase; TBARS: Thiobarbituric acid reactive substances.

### 7.2. Experimental Studies in Animal Models

In addition to these alternative therapies, other products have also been explored in GDM, including vegetal extracts, mainly aiming to assess the presence of bioactive compounds. However, special attention regarding toxicity must be considered since these products must be innocuous to maternal health, placental tissues, and cytotrophoblast [164,183]. In this regard, to be recognized as safe, these products with potential antioxidant and anti-inflammatory effects must be tested in animal models to provide scientific support for future clinical trials. Therefore, in addition to the studies listed above, researchers also investigated the use of natural products in induced GDM in animal models. Table 3 gathers these reports.

It was possible to identify that some of these natural products are closely related to the attenuation of inflammatory processes and oxidative stress, such as *Picralima nitida*, *Nauclea latifolia,* and *Oxytenanthera abyssinica* [184], besides the nano-resveratro-zinc oxide complex, encapsulated with chitosan [185], raw propolis of stingless bee (*Heterotrigona itama*) [186], and thymoquinone [187], while others were related only to antiglycidic and antiinsulinemic activity, such as *Hibiscus rosa-sinensis* [188], *Orthosiphon stamineus* [189], *Lentinus edodes* [190], and *Mentha piperita* [191].

*Picralima nitida, Nauclea latifolia*, and *Oxytenanthera abyssinica* are plants traditionally used in Africa by diabetic patients. *Picralima nitida* seeds have many properties, including antimicrobial, anti-inflammatory, analgesic, and antidiabetic properties, while the root and stem of *Nauclea latifolia* present antimalarial, antiepileptic, anxiolytic, analgesic, and antidiabetic activity. The leaves of *Oxytenanthera abyssinica* have a remarkable antidiabetic capacity. Their properties, in particular, antidiabetic properties, come from bioactive compounds present in these plants, such as polyphenols (tannins, flavonoids, anthocyanins, leucoanthocyanidins, and quinolone derivatives), which act through antioxidant mechanisms, such as inhibiting the phosphorylation of extracellular signal-regulated protein kinase (ERK) and p38 MAPK [184].

Resveratrol (3,5,4-trihydroxystilbene) is a polyphenolic compound of which the activities include preventing and attenuating insulin resistance. However, despite being a bioactive compound present in several natural products, its bioavailability may be compromised by several adjacent factors. Therefore, researchers developed a resveratrol–zinc complex and encapsulated it with chitosan nanoparticles to increase its bioavailability. It is noteworthy that both resveratrol and zinc are considered antioxidant agents for decreasing the expression of pro-inflammatory cytokines and RONS and for acting on insulin sensitivity. In pregnant rats induced to GDM, this complex presented antidiabetic activity by modulating IL-6 and MCP-1 and reduced some indicators of oxidative stress, such as GRP78, p-IRE1α, p-eIF2α, and pPERK. [185].

Propolis is a resinous substance collected by bees from the buds and exudates of plants in different world regions, currently considered a food product. Diverse kinds of propolis are known for their anti-inflammatory and antioxidant activities. In GDM, the antidiabetic activity of the propolis from *Heterotrigona itama* is closely related to the attenuation of pro-inflammatory cytokines and RONS. However, its use in pregnant women has some limitations. Its alcoholic extraction (which has a higher content of bioactive compounds) has been strongly discouraged. For this reason, despite the recognition of benefits, there is a risk, despite being low. The extraction strategies, aimed mainly at the safety of pregnant women, placental tissues, and cytotrophoblasts, should be considered [186].

**Table 3:** Alternative therapies used in animal models with induced GDM.

Source	Study Type	Animal Model	Diabetogenic Drug	Intervention	Main Findings
Yessoufou et al. (2013) [184]	Experimental study	Wistar rats	Streptozotocin (20 mg/kg)	I1: *Picralima nitida* (seeds); I2: *Nauclea latifolia* (root and stem); I3: *Oxytenanthera abyssinica* (leaves). All of these extracts were injected intraperitoneally at a concentration of 25 mg/kg (12–15 days)	Groups I1, I2, and I3 showed attenuation of GDM-induced hyperglycemia in pregnant rats. Among the three groups, the extract from group I1 exhibited ↑antioxidant capacity compared with the others.
Du et al. (2020) [185]	Experimental study	Wistar rats albinos	Streptozotocin (45 mg/kg)	Nano resveratrol-zinc oxide complex, encapsulated with chitosan (200 mg/kg), daily for 28 days	Supplementation: ↓serum glucose levels, maintained the lipid content compared with the control; ↓inflammatory factors (IL-6) and endoplasmic reticulum stress (GRP78, p-IRE1α, p-eIF2α, and p-PERK).
Usman et al. (2018) [186]	Experimental study	Sprague Dawley rats	Streptozotocin (60 mg/kg)	Propolis (*Heterotrigona itama*) orally: 300 mg/kg/day subcutaneously: 5.0 IU/kg/day	Treatment: ↓serum glucose and MDA levels, similar to the insulin-treated group.
Badr et al. (2013) [187]	Experimental study	Swiss albino mice	Streptozotocin (50 mg/kg)	Thymoquinone (20 mg/kg/day)	Supplementation significantly restored serum levels of glucose, insulin, reactive oxygen species, proinflammatory cytokines (IL-1β, IL-6, and TNF-α) and lipids, as well as lymphocyte proliferation in offspring.
Afiune et al. (2017) [188]	Experimental study	Wistar rats	Streptozotocin (40 mg/kg)	Oral aqueous extract of *Hibiscus rosa-sinensis* (flower) (100 mg/kg; 0–7 days/200 mg/kg; 8–14 days/400 mg/kg; 15–21 days)	Supplementation: ↑maternal and fetal weight, ↓atherogenic index and coronary artery risk index, while ↓preimplantation loss rate compared with the untreated diabetic group.
Lokman et al. (2019) [189]	Experimental study	Sprague Dawley rats	Streptozotocin (30 mg/kg)	*Orthosiphon stamineus* (0.1 g/100 g of peso)	Supplementation: significantly ↓serum glucose level. No mortality or signs of toxicity were recorded throughout the study, either for the female rat or her offspring.
Laurino et al. (2019) [190]	Experimental study	Wistar rats	Streptozotocin (40 mg/kg)	*Lentinus edodes* (100 mg/kg)	Supplementation: did not reduce hyperglycemia in rats with induced GDM, but ↑maternal insulin levels and ↓ALT, AST, triglyceride, and total cholesterol levels.
Barbalho et al. (2011) [191]	Experimental study	Wistar rats	Streptozotocin (40 mg/kg)	*Mentha piperita* (Peppermint) (Juice, 100 g/L)	Significantly ↓levels of glucose, cholesterol, LDL-c, and triglycerides and significantly ↑HDL-c levels.

I: Intervention; ↑: Increase; ↓: Decrease; IL-6: Interleukin 6; ALT: Alanine aminotransferase; AST: Aspartate aminotransferase; IL-1β: Interleukin 1β; IL-6: Interleukin 6; TNF-α: Tumor necrosis factor α; HDL-c: High-density cholesterol; LDL-c: Low-density lipoprotein cholesterol.

Thymoquinone is the biologically active compound of the black seed essential oil, which has numerous pharmacological properties, including antidiabetic, antioxidant, and anti-inflammatory activities. In animal models, thymoquinone showed positive results not only for rats but also for offspring to reduce the expression of pro-inflammatory pathways, cytokines, and RONS, reflecting a better organic general state [187]. However, despite the beneficial evidence regarding thymoquinone, some minimal side effects have been reported, such as weight loss, dyspnoea, signs of diffuse inflammation, and peritonitis [192].

However, despite the findings, further studies are needed to establish the standardization of the safety and toxicity threshold for the dose of these natural products and their extracts and solid waste. In this way, it is noteworthy to contribute not only to maternal and child health in the short and long terms but also, in a broad perspective, to identify an efficient therapy that can alleviate this critical public health problem in addition to favoring environmental sustainability and economic growth.

## 8. Limitations

The limitation of this study lies in some factors: First, although GDM is a complex metabolic disorder, with its first descriptions in the 60 s, so far there is no standardization and/or unification of cutoff points and conditions for its diagnosis and screening, a fact that limits data analysis, as studies adopt different cutoff points for the diagnosis of this disease. Additionally, one can point out the modulation of the intestinal microbiota, since it is currently known that intestinal dysbiosis is related to numerous diseases, and GDM can be included. However, studies are needed in order to identify the impacts on the GDM, and on the transgenerational colonization of children arising from these pregnancies as well as the identification of bacterial strains that can be included in the management of care in the GDM, aiming at mitigating the harmful impacts on maternal and fetal health. About alternative therapies, although there is a great recent interest in the scientific community, many of the results still have limitations, especially in their interpretation, in addition to having a risk of toxicity for pregnant women, placental tissues, and fetuses. As such, we strongly recommend conducting experimental studies and randomized clinical trials aimed at identifying a safe dose with protective effects.

## 9. Conclusions

GDM is a complex metabolic disorder that involves several etiological and pathophysiological factors, culminating in insulin resistance, accompanied by hyperglycemia, which can trigger a series of adverse outcomes in the health of the maternal-fetal binomial. Due to these highly serious repercussions, GDM can be considered a critical public health problem in the short and long terms, which requires urgent attention from public health policies and researchers in the maternal and child health area.

As for the pathophysiological aspects that establish the communication between inflammatory processes and nitroxidative stress in GDM, this review unveiled the molecular routes that involve these processes. It was also possible to identify a possible communication of the intestinal microbiota with the GDM, which in dysbiosis conditions, could contribute to the endogenous amplification of inflammatory and nitroxidative stress, being, therefore, an important therapeutic target in GDM, both preventively and for treatment, through the use of probiotics and prebiotics, individually or together (symbiotics).

In addition, recently, the scientific community has been looking into alternative or unconventional therapies for the treatment of GDM, mainly from natural products, due to the presence of bioactive compounds. However, despite having numerous benefits, these products can be toxic to pregnant women, placental tissues, and fetuses. Therefore, urgent designs are needed in order to identify safe and effective doses in attenuating inflammatory processes and oxidative stress, and consequently adverse maternal and perinatal outcomes.

## Abbreviations

ADA	American Diabetes Association
AGE	advanced glycation end products
ALA	α-linolenic acid
ALT	alanine aminotransferase
AST	aspartate aminotransferase
ATP	adenosine triphosphate
BAFF	B cell activating factor
BW/GA	relationship between BW and GA
BW	birth weight
CAT	catalase
CD40	cluster of differentiation 40
CDKAL1	CDK5 regulatory subunit-associated protein 1-like 1
CDKN2A/2B	cyclin-dependent kinase inhibitor 2A/2B
CFU	colony forming unit
CGRP	calcitonin gene-related peptide
CNCD	chronic non-communicable diseases
CRP	C-reactive protein
CVD	cardiovascular diseases
DASH	dietary approaches to stop hypertension
DHA	docosahexaenoic acid
DNA	deoxyribonucleic acid
EGCG	3-gallate epigallocatechin
EPA	eicosapentaenoic acid
ERK	extracellular signal-regulated protein kinase
FADH2	reduced flavin adenine dinucleotide
FBG	fasting blood glucose
FTO	fat mass- and obesity-associated gene
G6PD	glucose-6-phosphate dehydrogenase
GA	gestational age
GAPDH	glyceraldehyde-3-phosphate dehydrogenase
GDM	gestational diabetes mellitus
GH	growth hormone
GLUT	glucose transporters
GLUT-1	glucose transporter 1
GPCRs	G-protein-coupled receptors
GPx	glutathione peroxidase
GR	glutathione reductase
GRP78	78-kDa glucose-regulated protein
GSH	reduced glutathione
GSSG	reduces oxidized glutathione disulfide
GST	glutathione S-transferase
HAPO	hyperglycemia and adverse pregnancy outcomes
HDACs	histone deacetylases
HDL-c	high-density lipoprotein cholesterol
HHEX	hematopoietically expressed homeobox
HOMA-IR	homeostatic assessment model for insulin resistance
IADPSG	International Association of Diabetes in Pregnancy Study Group
IDF	International Diabetes Federation
IFGO	International Federation of Gynecology and Obstetrics
IFN-γ	interferon gama
IGF-1	type 1 insulin-like growth factor
IGF2BP2	insulin-like growth factor 2 MRNA binding protein 2
IKK	IκB kinase complex
IKKα	IKK alpha subunit
IKKβ	IKK beta subunit
IKKγ	IKK gamma subunit
IL-1β	interleukin 1β
IL-6	Interleukin 6
IRS	insulin receptor substrates
IκBs	inhibitory regulators of NF-Kb
JAK	Janus kinase pathway
LBW	low weight at birth
LDL-c	low-density lipoprotein cholesterol
LDLR	low-density lipoprotein receptor
LGA	large for gestational age
LP(a)	lipoprotein a
LPS	lipopolysaccharides
MCP-1	monocyte chemoattractant protein-1
MDA	malondialdehyde
MnSOD	manganese superoxide dismutase
MODY	maturity onset diabetes of the young
mTOR	mammalian target of rapamycin
MUFA	monounsaturated fatty acids
NAD^+^	nicotinamide adenine dinucleotide oxidized
NADH	nicotinamide adenine dinucleotide
NADP^+^	nicotinamide adenine dinucleotide phosphate oxidized
NADPH	nicotinamide adenine dinucleotide phosphate
NB	newborns
NDDG	National Diabetes Data Group
NF-Kb	nuclear factor kappa B
OGTT	Oral Glucose Tolerance Test
p38 MAPK	mitogen-activated protein kinase
PBMC	peripheral blood mononuclear cells
p-eIF2α	phosphorylation of eukaryotic translation initiation factor 2 alpha
PFOA	perfluorooctanoic acid
PI3K	phosphatidylinositol-3-kinase
p-IRE1α	phosphorylation of inositol-requiring enzyme-1alpha
PKB	protein kinase B
PKC	activation of protein kinase C
POS	polycystic ovary syndrome
PPARs	peroxisome proliferator-activated receptors
PPAR-γ	peroxisome proliferator
pPERK	phosphorylation of protein kinase R-like endoplasmic reticulum kinase
PTH	parathormone
PUFAS	polyunsaturated fatty acids
QUICKI	Quantitative Insulin Sensitivity Check Index
RAGE	receptor of AGE
RNA	ribonucleic acid
RNS	reactive nitrogen species
RONS	reactive oxygen and nitrogen species
ROS	reactive oxygen species
SCFA	short-chain fatty acids
SCL30A8	solute carrier family 30 member 8 *gene*
Ser727	Serine 727
SGA	small for gestational age
SOD	superoxide dismutase
SOSC3	suppressor of cytokine signaling 3
STAT	signal transducers and activators of transcription
STAT3	signal transducers and activators of transcription 3
T2DM	type 2 diabetes mellitus
TAC	total antioxidant capacity
TCA	tricarboxylic acid
TCF2	transcription factor 2
TCF7L2	transcription factor 7-like 2
TG	triglyceride
TLR-4	toll-like receptors type 4
TLRs	toll-like receptors
TMAO	trimethylamine N-oxide
TNF-α	tumor necrosis factor α
TNF-β	cytokines of the lymphotoxin family
Tyr705	tyrosine 705
Tyr727	tyrosine 727
VLDL-c	very low-density lipoprotein cholesterol
WHO	World Health Organization
ZO-1	zonulin
ω-3	omega-3

## References

[1] Lappas M., Hiden U., Desoye G., Froehlich J., Hauguel-de Mouzon S., Jawerbaum A. (2011). The role of oxidative stress in the pathophysiology of gestational diabetes mellitus. Antioxid. Redox Signal..

[2] Pantham P., Aye I.L., Powell T.L. (2015). Inflammation in maternal obesity and gestational diabetes mellitus. Placenta.

[3] Plows J.F., Stanley J.L., Baker P.N., Reynolds C.M., Vickers M.H. (2018). The Pathophysiology of Gestational Diabetes Mellitus. Int. J. Mol. Sci..

[4] McIntyre H.D., Catalano P., Zhang C., Desoye G., Mathiesen E.R., Damm P. (2019). Gestational diabetes mellitus. Nat. Rev. Dis. Primers.

[5] American Diabetes Association (2018). Classification and Diagnosis of Diabetes: Standards of Medical Care in Diabetes—2018. Diabetes Care.

[6] Szmuilowicz E.D., Josefson J.L., Metzger B.E. (2019). Gestational Diabetes Mellitus. Endocrinol. Metab. Clin. N. Am..

[7] Chiefari E., Arcidiacono B., Foti D., Brunetti A. (2017). Gestational diabetes mellitus: An updated overview. J. Endocrinol. Investig..

[8] Dirar A.M., Doupis J. (2017). Gestational diabetes from A to Z. World J. Diabetes.

[9] Li B.Y., Xu X.Y., Gan R.Y., Sun Q.C., Meng J.M., Shang A., Mao Q.Q., Li H.B. (2019). Targeting Gut Microbiota for the Prevention and Management of Diabetes Mellitus by Dietary Natural Products. Foods.

[10] Chen Q., Wu W., Yang H., Zhang P., Feng Y., Wang K., Wang Y., Wang S., Zhang Y. (2020). A Vegetable Dietary Pattern Is Associated with Lowered Risk of Gestational Diabetes Mellitus in Chinese Women. Diabetes Metab. J..

[11] Wei B., Wang S., Wang Y., Ke S., Jin W., Chen J., Zhang H., Sun J., Henning S.M., Wang J. (2021). Gut microbiota-mediated xanthine metabolism is associated with resistance to high-fat diet-induced obesity. J. Nutr. Biochem..

[12] Landete J.M., Arqués J., Medina M., Gaya P., de Las Rivas B., Muñoz R. (2016). Bioactivation of Phytoestrogens: Intestinal Bacteria and Health. Crit. Rev. Food Sci. Nutr..

[13] Zhu C., Yang H., Geng Q., Ma Q., Long Y., Zhou C., Chen M. (2015). Association of oxidative stress biomarkers with gestational diabetes mellitus in pregnant women: A case-control study. PLoS ONE.

[14] Sun C.C., Lai Y.N., Wang W.H., Xu X.M., Li X.Q., Wang H., Zheng J.Y., Zheng J.Q. (2020). Metformin Ameliorates Gestational Diabetes Mellitus-Induced Endothelial Dysfunction via Downregulation of p65 and Upregulation of Nrf2. Front. Pharmacol..

[15] Wang J., Zheng J., Shi W., Du N., Xu X., Zhang Y., Ji P., Zhang F., Jia Z., Wang Y. (2018). Dysbiosis of maternal and neonatal microbiota associated with gestational diabetes mellitus. Gut.

[16] Brawerman G.M., Dolinsky V.W. (2018). Therapies for gestational diabetes and their implications for maternal and offspring health: Evidence from human and animal studies. Pharmacol. Res..

[17] Ponzo V., Fedele D., Goitre I., Leone F., Lezo A., Monzeglio C., Finocchiaro C., Ghigo E., Bo S. (2019). Diet-Gut Microbiota Interactions and Gestational Diabetes Mellitus (GDM). Nutrients.

[18] American Diabetes Association (2021). 2. Classification and Diagnosis of Diabetes: Standards of Medical Care in Diabetes—2021. Diabetes Care.

[19] International Diabetes Federation (2017). IDF Diabetes Atlas.

[20] Organização Pan-Americana de Saúde, Ministério da Saúde, Federação Brasileira das Associações de Ginecologia e Obstetrícia, Sociedade Brasileira de Diabetes Rastreamento e Diagnóstico de Diabetes Mellitus Gestacional no Brasil—Brasília. 2017. E-book, pp. 15–19. https://www.febrasgo.org.br/images/pec/CNE_pdfs/Rastreamento-Diabetes.pdf.

[21] O’Sullivan J.B., Mahan C.M. (1964). Criteria for the oral glucose tolerance test in pregnancy. Diabetes.

[22] National Diabetes Data Group (1979). Classification and diagnosis of diabetes mellitus and other categories of glucose intolerance. National Diabetes Data Group. Diabetes.

[23] Carpenter M.W., Coustan D.R. (1982). Criteria for screening tests for gestational diabetes. Am. J. Obstet. Gynecol..

[24] American College of Obstetrics and Gynecology (1991). Proceedings of the Third International Workshop-Conference on Gestational Diabetes Mellitus. November 8–10,1990, Chicago, Illinois. Diabetes.

[25] HAPO Study Cooperative Research Group (2008). Hyperglycemia and adverse pregnancy outcomes. N. Engl. J. Med..

[26] World Health Organization (1999). Definition, Diagnosis and Classification of Diabetes Mellitus and its Complications.

[27] International Association of Diabetes and Pregnancy Study Groups Consensus Panel (2010). International association of diabetes and pregnancy study groups recommendations on the diagnosis and classification of hyperglycemia in pregnancy. Diabetes Care.

[28] Organização Pan-Americana da Saúde (2016). Rastreamento e Diagnóstico de Diabetes Mellitus Gestacional No Brasil. Ministério da Saúde. Federação Brasileira das Associações de Ginecologia e Obstetrícia. (DF): OPAS.

[29] HAPO Study Cooperative Research Group (2002). The Hyperglycemia and Adverse Pregnancy Outcome (HAPO) Study. Int. J. Gynaecol. Obstet..

[30] Blumer I., Hadar E., Hadden D.R., Jovanovic L., Mestman J.H., Murad M.H., Yogev Y. (2013). Diabetes and pregnancy: An endocrine society clinical practice guideline. J. Clin. Endocrinol. Metab..

[31] World Health Organization (2013). Diagnostic Criteria and Classification of Hyperglycaemia First Detected in Pregnancy: A World Health Organization Guideline.

[32] Hod M., Kapur A., Sacks D.A., Hadar E., Agarwal M., Di Renzo G.C., Roura L.C., McIntyre H.D., Morris J.L., Divakar H. (2015). The International Federation of Gynecology and Obstetrics (FIGO) initiative on gestational diabetes mellitus: A pragmatic guide for diagnosis, management, and care. Int. J. Gynaecol. Obstet..

[33] McIntyre H.D., Sacks D.A., Barbour L.A., Feig D.S., Catalano P.M., Damm P., McElduff A. (2016). Issues with the diagnosis and classification of hyperglycemia in early pregnancy. Diabetes Care.

[34] Schmidt M.I., Duncan B.B., Reichelt A.J., Branchtein L., Matos M.C., e Forti A.C., Spichler E.R., Pousada J.M., Teixeira M.M., Yamashita T. (2001). Gestational diabetes mellitus diagnosed with a 2-h 75-g oral glucose tolerance test and adverse pregnancy outcomes. Diabetes Care.

[35] Trujillo J., Vigo A., Reichelt A., Duncan B.B., Schmidt M.I. (2016). Fasting plasma glucose to avoid a full OGTT in the diagnosis of gestational diabetes. Diabetes Res. Clin. Pract..

[36] Chen P., Wang S., Ji J., Ge A., Chen C., Zhu Y., Xie N., Wang Y. (2016). Risk factors and management of gestational diabetes. Cell Biochem. Biophys..

[37] Zhang C., Rawal S., Chong Y.S. (2016). Risk factors for gestational diabetes: Is prevention possible?. Diabetologia.

[38] Carolan M. (2013). Maternal age ≥45 years and maternal and perinatal outcomes: A review of the evidence. Midwifery.

[39] Lean S.C., Derricott H., Jones R.L., Heazell A.E.P. (2017). Advanced maternal age and adverse pregnancy outcomes: A systematic review and meta-analysis. PLoS ONE.

[40] Cho Y.M., Kim T.H., Lim S., Choi S.H., Shin H.D., Lee H.K., Park K.S., Jang H.C. (2009). Type 2 diabetes-associated genetic variants discovered in the recent genome-wide association studies are related to gestational diabetes mellitus in the Korean population. Diabetologia.

[41] Zhou Y., Xie N., Zhang L., Chen D. (2021). Impact of family history of diabetes on blood glucose, lipid levels and perinatal outcomes in pregnant women with gestational diabetes mellitus. J. Zhejiang Univ..

[42] Lauenborg J., Grarup N., Damm P., Borch-Johnsen K., Jørgensen T., Pedersen O., Hansen T. (2009). Common type 2 diabetes risk gene variants associate with gestational diabetes. J. Clin. Endocrinol. Metab..

[43] Urbanová J., Brunerová L., Nunes M.A., Brož J. (2020). MODY diabetes and screening of gestational diabetes. Ceska Gynekol..

[44] Pu J., Zhao B., Wang E.J., Nimbal V., Osmundson S., Kunz L., Popat R.A., Chung S., Palaniappan L.P. (2015). Racial/Ethnic Differences in Gestational Diabetes Prevalence and Contribution of Common Risk Factors. Paediatr. Perinat. Epidemiol..

[45] Jaffe A., Giveon S., Rubin C., Novikov I., Ziv A., Kalter-Leibovici O. (2020). Gestational diabetes risk in a multi-ethnic population. Acta Diabetol..

[46] Schwartz N., Nachum Z., Green M.S. (2015). The prevalence of gestational diabetes mellitus recurrence—Effect of ethnicity and parity: A metaanalysis. Am. J. Obstet. Gynecol..

[47] Shang M., Dong X., Hou L. (2018). Correlation of adipokines and markers of oxidative stress in women with gestational diabetes mellitus and their newborns. J. Obstet. Gynaecol. Res..

[48] LifeCycle Project-Maternal Obesity and Childhood Outcomes Study Group (2019). Association of Gestational Weight Gain with Adverse Maternal and Infant Outcomes. JAMA.

[49] Barakat R., Refoyo I., Coteron J., Franco E. (2019). Exercise during pregnancy has a preventative effect on excessive maternal weight gain and gestational diabetes. A randomized controlled trial. Braz. J. Phys. Ther..

[50] Zhang C., Schulze M.B., Solomon C.G., Hu F.B. (2006). A prospective study of dietary pattRNS, meat intake and the risk of gestational diabetes mellitus. Diabetologia.

[51] Badon S.E., Wartko P.D., Qiu C., Sorensen T.K., Williams M.A., Enquobahrie D.A. (2016). Leisure Time Physical Activity and Gestational Diabetes Mellitus in the Omega Study. Med. Sci. Sports Exerc..

[52] Mijatovic-Vukas J., Capling L., Cheng S., Stamatakis E., Louie J., Cheung N.W., Markovic T., Ross G., Senior A., Brand-Miller J.C. (2018). Associations of Diet and Physical Activity with Risk for Gestational Diabetes Mellitus: A Systematic Review and Meta-Analysis. Nutrients.

[53] Lau C., Thibodeaux J.R., Hanson R.G., Narotsky M.G., Rogers J.M., Lindstrom A.B., Strynar M.J. (2006). Effects of perfluorooctanoic acid exposure during pregnancy in the mouse. Toxicol. Sci..

[54] Fenton S.E., Reiner J.L., Nakayama S.F., Delinsky A.D., Stanko J.P., Hines E.P., White S.S., Lindstrom A.B., Strynar M.J., Petropoulou S.E. (2009). Analysis of PFOA in dosed CD-1 mice. Part 2. Disposition of PFOA in tissues and fluids from pregnant and lactating mice and their pups. Reprod. Toxicol..

[55] Steenland K., Tinker S., Shankar A., Ducatman A. (2010). Association of perfluorooctanoic acid (PFOA) and perfluorooctane sulfonate (PFOS) with uric acid among adults with elevated community exposure to PFOA. Environ. Health Perspect..

[56] Zhang C., Sundaram R., Maisog J., Calafat A.M., Barr D.B., Buck Louis G.M. (2015). A prospective study of prepregnancy serum concentrations of perfluorochemicals and the risk of gestational diabetes. Fertil. Steril..

[57] Eriksen K.T., Raaschou-Nielsen O., McLaughlin J.K., Lipworth L., Tjønneland A., Overvad K., Sørensen M. (2013). Association between plasma PFOA and PFOS levels and total cholesterol in a middle-aged Danish population. PLoS ONE.

[58] Wang H., Yang J., Du H., Xu L., Liu S., Yi J., Qian X., Chen Y., Jiang Q., He G. (2018). Perfluoroalkyl substances, glucose homeostasis, and gestational diabetes mellitus in Chinese pregnant women: A repeat measurement-based prospective study. Environ. Int..

[59] Kim M.K., Han K., You S.Y., Kwon H.S., Yoon K.H., Lee S.H. (2020). Prepregnancy smoking and the risk of gestational diabetes requiring insulin therapy. Sci. Rep..

[60] Bar-Zeev Y., Haile Z.T., Chertok I.A. (2020). Association between Prenatal Smoking and Gestational Diabetes Mellitus. Obstet. Gynecol..

[61] Hinkle S.N., Bao W., Wu J., Sun Y., Ley S.H., Tobias D.K., Qian F., Rawal S., Zhu Y., Chavarro J.E. (2021). Association of Habitual Alcohol Consumption with Long-term Risk of Type 2 Diabetes Among Women With a History of Gestational Diabetes. JAMA Netw. Open.

[62] Elting M.W., Korsen T.J.M., Bezemer P.D., Schoemaker J. (2001). Prevalence of diabetes mellitus, hypertension and cardiac complaints in a follow-up study of a Dutch PCOS population. Hum. Reprod..

[63] Rojas J., Chávez-Castillo M., Bermúdez V. (2014). O papel da metformina nos distúrbios metabólicos durante a gravidez: Síndrome dos Ovários Policísticos e Diabetes Mellitus Gestacional. Int. J. Reprod. Med..

[64] Rizzo G., Garzon S., Fichera M., Panella M.M., Catena U., Schiattarella A., de Franciscis P., Vilos G., Tesarik J., Török P. (2019). Vitamin D and Gestational Diabetes Mellitus: Is There a Link?. Antioxidants.

[65] Wang L., Zhang C., Song Y., Zhang Z. (2020). Deficiência de vitamina D no soro e risco de diabetes mellitus gestacional: Uma meta-análise. Arch. Med. Sci..

[66] Barker D.J., Bull A.R., Osmond C., Simmonds S.J. (1990). Fetal and placental size and risk of hypertension in adult life. BMJ.

[67] Yan J., Yang H. (2014). Gestational diabetes mellitus, programing and epigenetics. J. Matern. Fetal Neonatal Med..

[68] Franzago M., Fraticelli F., Stuppia L., Vitacolonna E. (2019). Nutrigenetics, epigenetics and gestational diabetes: Consequences in mother and child. Epigenetics.

[69] Xia Q., Cai H., Xiang Y.B., Zhou P., Li H., Yang G., Jiang Y., Shu X.O., Zheng W., Xu W.H. (2019). Prospective cohort studies of birth weight and risk of obesity, diabetes, and hypertension in adulthood among the Chinese population. J. Diabetes.

[70] Mendonça E.L.S.S., de Lima Macêna M., Bueno N.B., de Oliveira A.C.M., Mello C.S. (2020). Premature birth, low birth weight, small for gestational age and chronic non-communicable diseases in adult life: A systematic review with meta-analysis. Early Hum. Dev..

[71] Kamana K.C., Shakya S., Zhang H. (2015). Gestational diabetes mellitus and macrosomia: A literature review. Ann. Nutr. Metab..

[72] Gascho C.L., Leandro D.M., e Silva T.R., Silva J.C. (2017). Predictors of cesarean delivery in pregnant women with gestational diabetes mellitus. Rev. Bras. Ginecol. Obstet..

[73] Pedersen J. (1954). Weight and length at birth of infants of diabetic mothers. Acta Endocrinol..

[74] Freinkel N. (1980). Banting Lecture 1980. Of pregnancy and progeny. Diabetes.

[75] Moore T.R. (2002). A comparison of amniotic fluid fetal pulmonary phospholipids in normal and diabetic pregnancy. Am. J. Obstet. Gynecol..

[76] McFarland L.V., Raskin M., Daling J.R., Benedetti T.J. (1986). Erb/Duchenne’s palsy: A consequence of fetal macrosomia and method of delivery. Obstet. Gynecol..

[77] Vaquero G., Ramos A., Martinez J.C., Valero P., Nunez-Enamorado N., Simon-De Las Heras R., Camacho-Salas A. (2017). Paralisis braquial obstetrica: Incidencia, seguimiento evolutivo y factores pronosticos [Obstetric brachial plexus palsy: Incidence, monitoring of progress and prognostic factors]. Rev. Neurol..

[78] Araújo Júnior E., Peixoto A.B., Zamarian A.C., Elito Júnior J., Tonni G. (2017). Macrosomia. Best Pract. Res. Clin. Obstet. Gynaecol..

[79] Silva L., Plösch T., Toledo F., Faas M.M., Sobrevia L. (2020). Adenosine kinase and cardiovascular fetal programming in gestational diabetes mellitus. Biochim. Biophys. Acta Mol. Basis Dis..

[80] Fonseca D.L. (2015). Morbidity and mortality in Brazil. Cad. Saúde Colet..

[81] Lowe W.L., Scholtens D.M., Lowe L.P., Kuang A., Nodzenski M., Talbot O., Catalano P.M., Linder B., Brickman W.J., Clayton P. (2018). Association of Gestational Diabetes with Maternal Disorders of Glucose Metabolism and Childhood Adiposity. JAMA.

[82] Prates T. Nutrição no Início da Vida, Epigenética e Prevenção das Doenças Crônicas não Transmissíveis: Uma Janela de Oportunidades para Pediatras/São Paulo: ILSI Brasil—International Life Sciences Institute do Brasil: São Paulo, Brasil, 2018. https://ilsibrasil.org/wp-content/uploads/sites/9/2019/02/Fasc%C3%ADculo-EPIGEN%C3%89TICA.pdf.

[83] Liu T., Zheng W., Wang L., Wang L., Zhang Y. (2020). TLR4/NF-κB Signaling Pathway Participates in the Protective Effects of Apocynin on Gestational Diabetes Mellitus Induced Placental Oxidative Stress and Inflammation. Reprod. Sci..

[84] Pasternak Y., Ohana M., Biron-Shental T., Cohen-Hagai K., Benchetrit S., Zitman-Gal T. (2020). Thioredoxin, thioredoxin interacting protein and transducer and activator of transcription 3 in gestational diabetes. Mol. Biol. Rep..

[85] Mitchell S., Vargas J., Hoffmann A. (2016). Signaling via the NFκB system. Wiley Interdiscip. Rev. Syst. Biol. Med..

[86] Kuzmicki M., Telejko B., Wawrusiewicz-Kurylonek N., Lipinska D., Pliszka J., Wilk J., Zielinska A., Skibicka J., Szamatowicz J., Kretowski A. (2013). The expression of genes involved in NF-κB activation in peripheral blood mononuclear cells of patients with gestational diabetes. Eur. J. Endocrinol..

[87] Lawrence T. (2009). The nuclear factor NF-kappaB pathway in inflammation. Cold Spring Harb. Perspect. Biol..

[88] Ghanim H., Aljada A., Daoud N., Deopurkar R., Chaudhuri A., Dandona P. (2007). Role of inflammatory mediators in the suppression of insulin receptor phosphorylation in circulating mononuclear cells of obese subjects. Diabetologia.

[89] Mello V.D., Kolehmainen M., Pulkkinen L., Schwab U., Mager U., Laaksonen D.E., Niskanen L., Gylling H., Atalay M., Rauramaa R. (2008). Downregulation of genes involved in NFkappaB activation in peripheral blood mononuclear cells after weight loss is associated with the improvement of insulin sensitivity in individuals with the metabolic syndrome: The GENOBIN study. Diabetologia.

[90] Abell S.K., De Courten B., Boyle J.A., Teede H.J. (2015). Inflammatory and Other Biomarkers: Role in Pathophysiology and Prediction of Gestational Diabetes Mellitus. Int. J. Mol. Sci..

[91] Nguyen-Ngo C., Jayabalan N., Salomon C., Lappas M. (2019). Molecular pathways disrupted by gestational diabetes mellitus. J. Mol. Endocrinol..

[92] Bossick A.S., Peters R.M., Burmeister C., Kakumanu N., Shill J.E., Cassidy-Bushrow A.E. (2016). Antenatal inflammation and gestational diabetes mellitus risk among pregnant African-American women. J. Reprod. Immunol..

[93] Loh C.Y., Arya A., Naema A.F., Wong W.F., Sethi G., Looi C.Y. (2019). Signal Transducer and Activator of Transcription (STATs) Proteins in Cancer and Inflammation: Functions and Therapeutic Implication. Front. Oncol..

[94] Gianotti T.F., Sookoian S., Gemma C., Burgueño A.L., González C.D., Pirola C.J. (2008). Study of genetic variation in the STAT3 on obesity and insulin resistance in male adults. Obesity.

[95] Recio C., Guerra B., Guerra-Rodríguez M., Aranda-Tavío H., Martín-Rodríguez P., de Mirecki-Garrido M., Brito-Casillas Y., García-Castellano J.M., Estévez-Braun A., Fernández-Pérez L. (2019). Signal transducer and activator of transcription (STAT)-5: An opportunity for drug development in oncohematology. Oncogene.

[96] Kim J.H., Kim J.E., Liu H.Y., Cao W., Chen J. (2008). Regulation of interleukin-6-induced hepatic insulin resistance by mammalian target of rapamycin through the STAT3-SOCS3 pathway. J. Biol. Chem..

[97] Heo Y.J., Choi S.E., Jeon J.Y., Han S.J., Kim D.J., Kang Y., Lee K.W., Kim H.J. (2019). Visfatin Induces Inflammation and Insulin Resistance via the NF-κB and STAT3 Signaling Pathways in Hepatocytes. J. Diabetes Res..

[98] Mashili F., Chibalin A.V., Krook A., Zierath J.R. (2013). Constitutive STAT3 phosphorylation contributes to skeletal muscle insulin resistance in type 2 diabetes. Diabetes.

[99] Gao A., Van Dyke T.E. (2014). Role of suppressors of cytokine signaling 3 in bone inflammatory responses. Front. Immunol..

[100] Kim T.H., Choi S.E., Ha E.S., Jung J.G., Han S.J., Kim H.J., Kim D.J., Kang Y., Lee K.W. (2013). IL-6 induction of TLR-4 gene expression via STAT3 has an effect on insulin resistance in human skeletal muscle. Acta Diabetol..

[101] Tan J., Xu J., Wei G., Zhang L., Sun L., Wang G., Li F., Jiang F. (2019). HNF1α Controls Liver Lipid Metabolism and Insulin Resistance via Negatively Regulating the SOCS-3-STAT3 Signaling Pathway. J. Diabetes Res..

[102] Zhang L., Chen Z., Wang Y., Tweardy D.J., Mitch W.E. (2020). Stat3 activation induces insulin resistance via a muscle-specific E3 ubiquitin ligase Fbxo40. Am. J. Physiol. Endocrinol. Metab..

[103] Nourbakhsh M., Nourbakhsh M., Gholinejad Z., Razzaghy-Azar M. (2015). Visfatin in obese children and adolescents and its association with insulin resistance and metabolic syndrome. Scand. J. Clin. Lab. Investig..

[104] Radzicka S., Pietryga M., Iciek R., Brązert J. (2018). The role of visfatin in pathogenesis of gestational diabetes (GDM). Ginekol. Pol..

[105] Decker T., Kovarik P. (2000). Serine phosphorylation of STATs. Oncogene.

[106] Reyna S.M., Ghosh S., Tantiwong P., Meka C.S., Eagan P., Jenkinson C.P., Cersosimo E., Defronzo R.A., Coletta D.K., Sriwijitkamol A. (2008). Elevated toll-like receptor 4 expression and signaling in muscle from insulin-resistant subjects. Diabetes.

[107] Sies H., Berndt C., Jones D.P. (2017). Oxidative Stress. Annu. Rev. Biochem..

[108] Al-Shehri S.S. (2021). Reactive oxygen and nitrogen species and innate immune response. Biochimie.

[109] Gauster M., Majali-Martinez A., Maninger S., Gutschi E., Greimel P.H., Ivanisevic M., Djelmis J., Desoye G., Hiden U. (2017). Maternal Type 1 diabetes activates stress response in early placenta. Placenta.

[110] Tenório M.B., Ferreira R.C., Moura F.A., Bueno N.B., de Oliveira A.C.M., Goulart M.O.F. (2019). Cross-Talk between Oxidative Stress and Inflammation in Preeclampsia. Oxid. Med. Cell. Longev..

[111] Taysi S., Tascan A.S., Ugur M.G., Demir M. (2019). Radicals, Oxidative/Nitrosative Stress and Preeclampsia. Mini Rev. Med. Chem..

[112] Marrocco I., Altieri F., Peluso I. (2017). Measurement and Clinical Significance of Biomarkers of Oxidative Stress in Humans. Oxid. Med. Cell. Longev..

[113] Hastie R., Lappas M. (2014). The effect of pre-existing maternal obesity and diabetes on placental mitochondrial content and electron transport chain activity. Placenta.

[114] Vidal Z.E., Rufino S.C., Tlaxcalteco E.H., Trejo C.H., Campos R.M., Meza M.N., Rodríguez R.C., Arroyo-Helguera O. (2014). Oxidative stress increased in pregnant women with iodine deficiency. Biol. Trace Elem. Res..

[115] Duan Y., Sun F., Que S., Li Y., Yang S., Liu G. (2018). Prepregnancy maternal diabetes combined with obesity impairs placental mitochondrial function involving Nrf2/ARE pathway and detrimentally alters metabolism of offspring. Obes. Res. Clin. Pract..

[116] Ramiro-Cortijo D., Herrera T., Rodríguez-Rodríguez P., López De Pablo Á.L., De La Calle M., López-Giménez M.R., Mora-Urda A.I., Gutiérrez-Arzapalo P.Y., Gómez-Rioja R., Aguilera Y. (2016). Maternal plasma antioxidant status in the first trimester of pregnancy and development of obstetric complications. Placenta.

[117] Agarwal A., Gupta S., Sharma R.K. (2005). Role of oxidative stress in female reproduction. Reprod. Biol. Endocrinol..

[118] Karamali M., Dadkhah F., Sadrkhanlou M., Jamilian M., Ahmadi S., Tajabadi-Ebrahimi M., Jafari P., Asemi Z. (2016). Effects of probiotic supplementation on glycaemic control and lipid profiles in gestational diabetes: A randomized, double-blind, placebo-controlled trial. Diabetes Metab..

[119] Papachristoforou E., Lambadiari V., Maratou E., Makrilakis K. (2020). Association of Glycemic Indices (Hyperglycemia, Glucose Variability, and Hypoglycemia) with Oxidative Stress and Diabetic Complications. J. Diabetes Res..

[120] Ighodaro O.M. (2018). Molecular pathways associated with oxidative stress in diabetes mellitus. Biomed. Pharmacother..

[121] Frijhoff J., Winyard P.G., Zarkovic N., Davies S.S., Stocker R., Cheng D., Knight A.R., Taylor E.L., Oettrich J., Ruskovska T. (2015). Clinical Relevance of Biomarkers of Oxidative Stress. Antioxid. Redox Signal..

[122] Belfiore A., Malaguarnera R., Vella V., Lawrence M.C., Sciacca L., Frasca F., Morrione A., Vigneri R. (2017). Insulin Receptor Isoforms in Physiology and Disease: An Updated View. Endocr. Rev..

[123] Volpe C.M.O., Villar-Delfino P.H., Dos Anjos P.M.F., Nogueira-Machado J.A. (2018). Cellular death, reactive oxygen species (ROS) and diabetic complications. Cell Death Dis..

[124] Sánchez-Santos A., Martínez-Hernández M.G., Contreras-Ramos A., Ortega-Camarillo C., Baiza-Gutman L.A. (2018). Hyperglycemia-induced mouse trophoblast spreading is mediated by reactive oxygen species. Mol. Reprod. Dev..

[125] Chiarello D.I., Abad C., Rojas D., Toledo F., Vázquez C.M., Mate A., Sobrevia L., Marín R. (2020). Oxidative stress: Normal pregnancy versus preeclampsia. Biochim. Biophys. Acta Mol. Basis Dis..

[126] Little B.D., Hopkins R.Z. (2020). Superoxide Dismutases in Biology and Medicine: Essentials and Recent Advances. React. Oxyg. Species.

[127] Giustarini D., Tsikas D., Colombo G., Milzani A., Dalle-Donne I., Fanti P., Rossi R. (2016). Pitfalls in the analysis of the physiological antioxidant glutathione (GSH) and its disulfide (GSSG) in biological samples: An elephant in the room. J. Chromatogr. B Analyt. Technol. Biomed. Life Sci..

[128] Bartolini D., Giustarini D., Pietrella D., Rossi R., Galli F. (2020). Glutathione S-transferase P influences the Nrf2-dependent response of cellular thiols to seleno-compounds. Cell Biol. Toxicol..

[129] Romo A.I.B., Dibo V.S., Abreu D.S., Carepo M.S.P., Neira A.C., Castillo I., Lemus L., Nascimento O.R., Bernhardt P.V., Sousa E.H.S. (2019). Ascorbyl and hydroxyl radical generation mediated by a copper complex adsorbed on gold. Dalton Trans..

[130] Di Vincenzo A., Tana C., El Hadi H., Pagano C., Vettor R., Rossato M. (2019). Antioxidant, Anti-Inflammatory, and Metabolic Properties of Tocopherols and Tocotrienols: Clinical Implications for Vitamin E Supplementation in Diabetic Kidney Disease. Int. J. Mol. Sci..

[131] Halliwell B., Gutteridge J.M.C. (2015). Free Radical in Biology and Medicine.

[132] Canfora E.E., Jocken J.W., Blaak E.E. (2015). Short-chain fatty acids in control of body weight and insulin sensitivity. Nat. Rev. Endocrinol..

[133] Clarke G., Stilling R.M., Kennedy P.J., Stanton C., Cryan J.F., Dinan T.G. (2014). Minireview: Gut microbiota: The neglected endocrine organ. Mol. Endocrinol..

[134] Meijnikman A.S., Gerdes V.E., Nieuwdorp M., Herrema H. (2018). Evaluating Causality of Gut Microbiota in Obesity and Diabetes in Humans. Endocr. Rev..

[135] Sircana A., Framarin L., Leone N., Berrutti M., Castellino F., Parente R., De Michieli F., Paschetta E., Musso G. (2018). Altered Gut Microbiota in Type 2 Diabetes: Just a Coincidence?. Curr. Diab. Rep..

[136] Hu C., Wong F.S., Wen L. (2015). Type 1 diabetes and gut microbiota: Friend or foe?. Pharmacol. Res..

[137] Crusell M.K.W., Hansen T.H., Nielsen T., Allin K.H., Rühlemann M.C., Damm P., Vestergaard H., Rørbye C., Jørgensen N.R., Christiansen O.B. (2018). Gestational diabetes is associated with change in the gut microbiota composition in third trimester of pregnancy and postpartum. Microbiome.

[138] Serino M., Fernández-Real J.M., García-Fuentes E., Queipo-Ortuño M., Moreno-Navarrete J.M., Sánchez A., Burcelin R., Tinahones F. (2013). The gut microbiota profile is associated with insulin action in humans. Acta Diabetol..

[139] Crommen S., Simon M.C. (2017). Microbial Regulation of Glucose Metabolism and Insulin Resistance. Genes.

[140] Bao W., Bowers K., Tobias D.K., Olsen S.F., Chavarro J., Vaag A., Kiely M., Zhang C. (2014). Prepregnancy low-carbohydrate dietary pattern and risk of gestational diabetes mellitus: A prospective cohort study. Am. J. Clin. Nutr..

[141] Mokkala K., Tertti K., Rönnemaa T., Vahlberg T., Laitinen K. (2017). Evaluation of serum zonulin for use as an early predictor for gestational diabetes. Nutr. Diabetes.

[142] Jayashree B., Bibin Y.S., Prabhu D., Shanthirani C.S., Gokulakrishnan K., Lakshmi B.S., Mohan V., Balasubramanyam M. (2014). Increased circulatory levels of lipopolysaccharide (LPS) and zonulin signify novel biomarkers of proinflammation in patients with type 2 diabetes. Mol. Cell. Biochem..

[143] Cortez R.V., Taddei C.R., Sparvoli L.G., Ângelo A.G.S., Padilha M., Mattar R., Daher S. (2019). Microbiome and its relation to gestational diabetes. Endocrine.

[144] Hasain Z., Mokhtar N.M., Kamaruddin N.A., Mohamed Ismail N.A., Razalli N.H., Gnanou J.V., Raja Ali R.A. (2020). Gut Microbiota and Gestational Diabetes Mellitus: A Review of Host-Gut Microbiota Interactions and Their Therapeutic Potential. Front. Cell. Infect. Microbiol..

[145] Ferrocino I., Ponzo V., Gambino R., Zarovska A., Leone F., Monzeglio C., Goitre I., Rosato R., Romano A., Grassi G. (2018). Changes in the gut microbiota composition during pregnancy in patients with gestational diabetes mellitus (GDM). Sci. Rep..

[146] Hill C., Guarner F., Reid G., Gibson G.R., Merenstein D.J., Pot B., Morelli L., Canani R.B., Flint H.J., Salminen S. (2014). Expert consensus document. The International Scientific Association for Probiotics and Prebiotics consensus statement on the scope and appropriate use of the term probiotic. Nat. Rev. Gastroenterol. Hepatol..

[147] Gomes A.C., Bueno A.A., de Souza R.G., Mota J.F. (2014). Gut microbiota, probiotics and diabetes. Nutr. J..

[148] Yan F., Cao H., Cover T.L., Whitehead R., Washington M.K., Polk D.B. (2007). Soluble proteins produced by probiotic bacteria regulate intestinal epithelial cell survival and growth. Gastroenterology.

[149] Banan A., Keshavarzian A., Zhang L., Shaikh M., Forsyth C.B., Tang Y., Fields J.Z. (2007). NF-kappaB activation as a key mechanism in ethanol-induced disruption of the F-actin cytoskeleton and monolayer barrier integrity in intestinal epithelium. Alcohol.

[150] Hajifaraji M., Jahanjou F., Abbasalizadeh F., Aghamohammadzadeh N., Abbasi M.M., Dolatkhah N. (2018). Effect of probiotic supplements in women with gestational diabetes mellitus on inflammation and oxidative stress biomarkers: A randomized clinical trial. Asia Pac. J. Clin. Nutr..

[151] Kinalski M., Sledziewski A., Telejko B., Zarzycki W., Kinalska I. (2000). Lipid peroxidation and scavenging enzyme activity in streptozotocin-induced diabetes. Acta Diabetol..

[152] Fugmann M., Breier M., Rottenkolber M., Banning F., Ferrari U., Sacco V., Grallert H., Parhofer K.G., Seissler J., Clavel T. (2015). The stool microbiota of insulin resistant women with recent gestational diabetes, a high risk group for type 2 diabetes. Sci. Rep..

[153] Kijmanawat A., Panburana P., Reutrakul S., Tangshewinsirikul C. (2019). Effects of probiotic supplements on insulin resistance in gestational diabetes mellitus: A double-blind randomized controlled trial. J. Diabetes Investig..

[154] Babadi M., Khorshidi A., Aghadavood E., Samimi M., Kavossian E., Bahmani F., Mafi A., Shafabakhsh R., Satari M., Asemi Z. (2019). The Effects of Probiotic Supplementation on Genetic and Metabolic Profiles in Patients with Gestational Diabetes Mellitus: A Randomized, Double-Blind, Placebo-Controlled Trial. Probiotics Antimicrob. Proteins.

[155] Badehnoosh B., Karamali M., Zarrati M., Jamilian M., Bahmani F., Tajabadi-Ebrahimi M., Jafari P., Rahmani E., Asemi Z. (2018). The effects of probiotic supplementation on biomarkers of inflammation, oxidative stress and pregnancy outcomes in gestational diabetes. J. Matern. Fetal Neonatal Med..

[156] Nabhani Z., Hezaveh S.J.G., Razmpoosh E., Asghari-Jafarabadi M., Gargari B.P. (2018). The effects of symbiotic supplementation on insulin resistance/sensitivity, lipid profile and total antioxidant capacity in women with gestational diabetes mellitus: A randomized double blind placebo controlled clinical trial. Diabetes Res. Clin. Pract..

[157] Jamilian M., Amirani E., Asemi Z. (2019). The effects of vitamin D and probiotic co-supplementation on glucose homeostasis, inflammation, oxidative stress and pregnancy outcomes in gestational diabetes: A randomized, double-blind, placebo-controlled trial. Clin. Nutr..

[158] Karamali M., Nasiri N., Taghavi Shavazi N., Jamilian M., Bahmani F., Tajabadi-Ebrahimi M., Asemi Z. (2018). The Effects of Synbiotic Supplementation on Pregnancy Outcomes in Gestational Diabetes. Probiotics Antimicrob. Proteins.

[159] Ahmadi S., Jamilian M., Tajabadi-Ebrahimi M., Jafari P., Asemi Z. (2016). The effects of synbiotic supplementation on markers of insulin metabolism and lipid profiles in gestational diabetes: A randomised, double-blind, placebo-controlled trial. Br. J. Nutr..

[160] Jafarnejad S., Saremi S., Jafarnejad F., Arab A. (2016). Effects of a Multispecies Probiotic Mixture on Glycemic Control and Inflammatory Status in Women with Gestational Diabetes: A Randomized Controlled Clinical Trial. J. Nutr. Metab..

[161] Dolatkhah N., Hajifaraji M., Abbasalizadeh F., Aghamohammadzadeh N., Mehrabi Y., Abbasi M.M. (2015). Is there a value for probiotic supplements in gestational diabetes mellitus? A randomized clinical trial. J. Health Popul. Nutr..

[162] Lindsay K.L., Brennan L., Kennelly M.A., Maguire O.C., Smith T., Curran S., Coffey M., Foley M.E., Hatunic M., Shanahan F. (2015). Impact of probiotics in women with gestational diabetes mellitus on metabolic health: A randomized controlled trial. Am. J. Obstet. Gynecol..

[163] Guo L., Ma J., Tang J., Hu D., Zhang W., Zhao X. (2019). Comparative Efficacy and Safety of Metformin, Glyburide, and Insulin in Treating Gestational Diabetes Mellitus: A Meta-Analysis. J. Diabetes Res..

[164] Koh L.M., Percival B., Pauley T., Pathak S. (2019). Complementary therapy and alternative medicine: Effects on induction of labour and pregnancy outcome in low risk post-dates women. Heliyon.

[165] Karamali M., Dastyar F., Badakhsh M.H., Aghadavood E., Amirani E., Asemi Z. (2020). The Effects of Selenium Supplementation on Gene Expression Related to Insulin and Lipid Metabolism, and Pregnancy Outcomes in Patients with Gestational Diabetes Mellitus: A Randomized, Double-Blind, Placebo-Controlled Trial. Biol. Trace Elem. Res..

[166] Gomez Ribot D., Diaz E., Fazio M.V., Gómez H.L., Fornes D., Macchi S.B., Gresta C.A., Capobianco E., Jawerbaum A. (2020). An extra virgin olive oil-enriched diet improves maternal, placental, and cord blood parameters in GDM pregnancies. Diabetes Metab. Res. Rev..

[167] Jamilian M., Samimi M., Mirhosseini N., Afshar Ebrahimi F., Aghadavod E., Taghizadeh M., Asemi Z. (2018). A Randomized Double-Blinded, Placebo-Controlled Trial Investigating the Effect of Fish Oil Supplementation on Gene Expression Related to Insulin Action, Blood Lipids, and Inflammation in Gestational Diabetes Mellitus-Fish Oil Supplementation and Gestational Diabetes. Nutrients.

[168] Fei B.B., Ling L., Hua C., Ren S.Y. (2014). Effects of soybean oligosaccharides on antioxidant enzyme activities and insulin resistance in pregnant women with gestational diabetes mellitus. Food Chem..

[169] Yuan L.J., Qin Y., Wang L., Zeng Y., Chang H., Wang J., Wang B., Wan J., Chen S.H., Zhang Q.Y. (2016). Capsaicin-containing chili improved postprandial hyperglycemia, hyperinsulinemia, and fasting lipid disorders in women with gestational diabetes mellitus and lowered the incidence of large-for-gestational-age newborns. Clin. Nutr..

[170] Sun X., Sun H., Zhang J., Ji X. (2016). Artemisia Extract Improves Insulin Sensitivity in Women With Gestational Diabetes Mellitus by Up-Regulating Adiponectin. J. Clin. Pharmacol..

[171] Asemi Z., Samimi M., Tabassi Z., Sabihi S.S., Esmaillzadeh A. (2013). A randomized controlled clinical trial investigating the effect of DASH diet on insulin resistance, inflammation, and oxidative stress in gestational diabetes. Nutrition.

[172] Karamali M., Bahramimoghadam S., Sharifzadeh F., Asemi Z. (2018). Magnesium-zinc-calcium-vitamin D co-supplementation improves glycemic control and markers of cardiometabolic risk in gestational diabetes: A randomized, double-blind, placebo-controlled trial. Appl. Physiol. Nutr. Metab..

[173] Jamilian M., Karamali M., Taghizadeh M., Sharifi N., Jafari Z., Memarzadeh M.R., Mahlouji M., Asemi Z. (2016). Vitamin D and Evening Primrose Oil Administration Improve Glycemia and Lipid Profiles in Women with Gestational Diabetes. Lipids.

[174] Hajimoosayi F., Jahanian Sadatmahalleh S., Kazemnejad A., Pirjani R. (2020). Effect of ginger on the blood glucose level of women with gestational diabetes mellitus (GDM) with impaired glucose tolerance test (GTT): A randomized double-blind placebo-controlled trial. BMC Complement. Med. Ther..

[175] Asemi Z., Karamali M., Esmaillzadeh A. (2014). Effects of calcium-vitamin D co-supplementation on glycaemic control, inflammation and oxidative stress in gestational diabetes: A randomised placebo-controlled trial. Diabetologia.

[176] Gunasegaran P., Tahmina S., Daniel M., Nanda S.K. (2021). Role of vitamin D-calcium supplementation on metabolic profile and oxidative stress in gestational diabetes mellitus: A randomized controlled trial. J. Obstet. Gynaecol. Res..

[177] Ostadmohammadi V., Samimi M., Mobini M., Zarezade Mehrizi M., Aghadavod E., Chamani M., Dastorani M., Asemi Z. (2019). The effect of zinc and vitamin E cosupplementation on metabolic status and its related gene expression in patients with gestational diabetes. J. Matern. Fetal Neonatal Med..

[178] Yang S., Lin R., Si L., Li Z., Jian W., Yu Q., Jia Y. (2019). Cod-Liver Oil Improves Metabolic Indices and hs-CRP Levels in Gestational Diabetes Mellitus Patients: A Double-Blind Randomized Controlled Trial. J. Diabetes Res..

[179] Jamilian M., Tabassi Z., Reiner Ž., Panahandeh I., Naderi F., Aghadavod E., Amirani E., Taghizadeh M., Shafabakhsh R., Satari M. (2020). The effects of n-3 fatty acids from flaxseed oil on genetic and metabolic profiles in patients with gestational diabetes mellitus: A randomised, double-blind, placebo-controlled trial. Br. J. Nutr..

[180] Asemi Z., Jamilian M., Mesdaghinia E., Esmaillzadeh A. (2015). Effects of selenium supplementation on glucose homeostasis, inflammation, and oxidative stress in gestational diabetes: Randomized, double-blind, placebo-controlled trial. Nutrition.

[181] Gao F., Wang G., Wang L., Guo N. (2017). Phytosterol nutritional supplement improves pregnancy and neonatal complications of gestational diabetes mellitus in a double-blind and placebo-controlled clinical study. Food Funct..

[182] Zhang H., Su S., Yu X., Li Y. (2017). Dietary epigallocatechin 3-gallate supplement improves maternal and neonatal treatment outcome of gestational diabetes mellitus: A double-blind randomised controlled trial. J. Hum. Nutr. Diet..

[183] Chandrasekhar D., Jose S.M., Jomy A., Joseph A., Pradeep A., Geoji A.S. (2018). Antiglycation property of passiflora edulis f. Flavicarpa deg. foliage in type 2 diabetic patients. Clin. Epidemiol. Glob. Health.

[184] Yessoufou A., Gbenou J., Grissa O., Hichami A., Simonin A.M., Tabka Z., Moudachirou M., Moutairou K., Khan N.A. (2013). Anti-hyperglycemic effects of three medicinal plants in diabetic pregnancy: Modulation of T cell proliferation. BMC Complement. Altern. Med..

[185] Du S., Lv Y., Li N., Huang X., Liu X., Li H., Wang C., Jia Y.F. (2020). Biological investigations on therapeutic effect of chitosan encapsulated nano resveratrol against gestational diabetes mellitus rats induced by streptozotocin. Drug Deliv..

[186] Usman U.Z., Bakar A.B.A., Mohamed M. (2018). Propolis improves pregnancy outcomes and placental oxidative stress status in streptozotocin-induced diabetic rats. BMC Complement. Altern. Med..

[187] Badr G., Mahmoud M.H., Farhat K., Waly H., Al-Abdin O.Z., Rabah D.M. (2013). Maternal supplementation of diabetic mice with thymoquinone protects their offspring from abnormal obesity and diabetes by modulating their lipid profile and free radical production and restoring lymphocyte proliferation via PI3K/AKT signaling. Lipids Health Dis..

[188] Afiune L.A.F., Leal-Silva T., Sinzato Y.K., Moraes-Souza R.Q., Soares T.S., Campos K.E., Fujiwara R.T., Herrera E., Damasceno D.C., Volpato G.T. (2017). Beneficial effects of Hibiscus rosa-sinensis, L. flower aqueous extract in pregnant rats with diabetes. PLoS ONE.

[189] Lokman E.F., Saparuddin F., Muhammad H., Omar M.H., Zulkapli A. (2019). Orthosiphon stamineus as a potential antidiabetic drug in maternal hyperglycemia in streptozotocin-induced diabetic rats. Integr. Med. Res..

[190] Laurino L.F., Viroel F.J.M., Caetano E., Spim S., Pickler T.B., Rosa-Castro R.M., Vasconcelos E.A., Jozala A.F., Hataka A., Grotto D. (2019). Lentinus edodes Exposure before and after Fetus Implantation: Materno-Fetal Development in Rats with Gestational Diabetes Mellitus. Nutrients.

[191] Barbalho S.M., Damasceno D.C., Spada A.P., da Silva V.S., Martuchi K.A., Oshiiwa M., Machado F.M., Mendes C.G. (2011). Metabolic Profile of Offspring from Diabetic Wistar Rats Treated with Mentha piperita (Peppermint). Evid. Based Complement. Alternat. Med..

[192] Abukhader M.M. (2012). The effect of route of administration in thymoquinone toxicity in male and female rats. Indian J. Pharm. Sci..

